# Early detection of autism using digital behavioral phenotyping

**DOI:** 10.1038/s41591-023-02574-3

**Published:** 2023-10-02

**Authors:** Sam Perochon, J. Matias Di Martino, Kimberly L. H. Carpenter, Scott Compton, Naomi Davis, Brian Eichner, Steven Espinosa, Lauren Franz, Pradeep Raj Krishnappa Babu, Guillermo Sapiro, Geraldine Dawson

**Affiliations:** 1 Department of Electrical and Computer Engineering, Duke University, Durham, NC USA; 2 Ecole Normale Supérieure Paris-Saclay, Gif-sur-Yvette, France; 3 Department of Psychiatry and Behavioral Sciences, Duke University, Durham, NC USA; 4 Duke Center for Autism and Brain Development, Duke University, Durham, NC USA; 5 Department of Pediatrics, Duke University, Durham, NC USA; 6 Office of Information Technology, Duke University, Durham, NC USA; 7 Duke Global Health Institute, Duke University, Durham, NC USA; 8 Departments of Biomedical Engineering, Mathematics, and Computer Science, Duke University, Durham, NC USA

**Keywords:** Disability, Machine learning

## Abstract

Early detection of autism, a neurodevelopmental condition associated with challenges in social communication, ensures timely access to intervention. Autism screening questionnaires have been shown to have lower accuracy when used in real-world settings, such as primary care, as compared to research studies, particularly for children of color and girls. Here we report findings from a multiclinic, prospective study assessing the accuracy of an autism screening digital application (app) administered during a pediatric well-child visit to 475 (17–36 months old) children (269 boys and 206 girls), of which 49 were diagnosed with autism and 98 were diagnosed with developmental delay without autism. The app displayed stimuli that elicited behavioral signs of autism, quantified using computer vision and machine learning. An algorithm combining multiple digital phenotypes showed high diagnostic accuracy with the area under the receiver operating characteristic curve = 0.90, sensitivity = 87.8%, specificity = 80.8%, negative predictive value = 97.8% and positive predictive value = 40.6%. The algorithm had similar sensitivity performance across subgroups as defined by sex, race and ethnicity. These results demonstrate the potential for digital phenotyping to provide an objective, scalable approach to autism screening in real-world settings. Moreover, combining results from digital phenotyping and caregiver questionnaires may increase autism screening accuracy and help reduce disparities in access to diagnosis and intervention.

## Abstract (web summary)

In a prospective study, an autism screening app administered using a tablet computer to young children showed high diagnostic accuracy and may facilitate a scalable approach to autism screening.

## Main

Autism spectrum disorder (ASD; henceforth ‘autism’) is a neurodevelopmental condition associated with challenges in social communication abilities and the presence of restricted and repetitive behaviors. Autism signs emerge between 9 and 18 months and include reduced attention to people, lack of response to name, differences in affective engagement and expressions and motor delays, among other features^1^. Commonly, children are screened for autism at their 18–24-month well-child visits using a parent questionnaire, the Modified Checklist for Autism in Toddlers-Revised with Follow-Up (M-CHAT-R/F)^2^. The M-CHAT-R/F has been shown to have higher accuracy in research settings^3^ compared to real-world settings, such as primary care, particularly for girls and children of color^4–7^. This is, in part, due to low rates of completion of the follow-up interview by pediatricians^8^. A study of >25,000 children screened in primary care found that the M-CHAT/F’s specificity was high (95.0%) but sensitivity was poor (39.0%), and its positive predictive value (PPV) was 14.6% (ref. ^6^). Thus, there is a need for accurate, objective and scalable autism screening tools to increase the accuracy of autism screening and reduce disparities in access to early diagnosis and intervention, which can improve outcomes^9^.

A promising screening approach is the use of eye-tracking technology to measure children’s attentional preferences for social versus nonsocial stimuli^10^. Autism is characterized by reduced spontaneous visual attention to social stimuli^10^. Studies of preschool and school-age children using machine learning (ML) of eye-tracking data reported encouraging findings for the use of eye-tracking for distinguishing autistic and neurotypical children^11,12^. However, because autism has a heterogeneous presentation involving multiple behavioral signs, eye-tracking tests alone may be insufficient as an autism screening tool. When an eye-tracking measure of social attention was used for autism screening in 1,863 (12–48 months old) children, the eye-tracking task had strong specificity (98.0%) but poor sensitivity (17.0%). The authors conclude that the eye-tracking task is useful for detecting a subtype of autism^13^.

By quantifying multiple autism-related behaviors, it may be possible to better capture the complex and variable presentation of autism reflected in current diagnostic assessments. Digital phenotyping can detect differences between autistic and neurotypical children in gaze patterns, head movements, facial expressions and motor behaviors^14–18^. We developed an application (app), SenseToKnow, which is administered on a tablet and displays brief, strategically designed movies while the child’s behavioral responses are recorded via the frontal camera embedded in the device. The movies are designed to elicit a wide range of autism-related behaviors, including social attention, facial expressions, head movements, response to name, blink rate and motor behaviors, which are quantified via computer vision analysis (CVA)^19–25^. ML is used to integrate multiple digital phenotypes into a combined algorithm that classifies children as autistic versus nonautistic and to generate metrics reflecting the quality of the app administration and confidence level associated with the diagnostic classification.

## Results

**Fig. 1:**
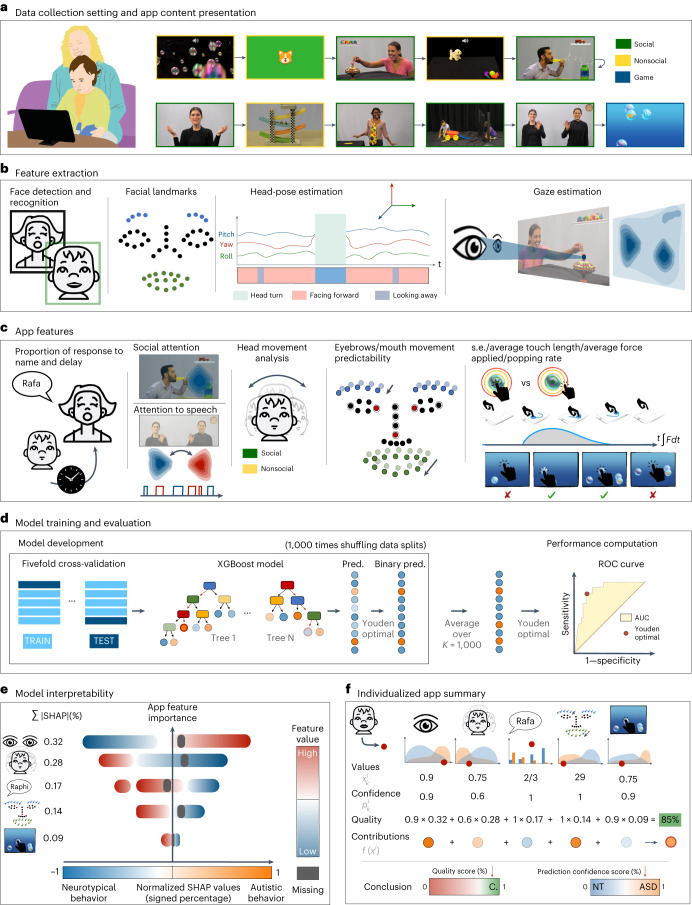
The SenseToKnow app workflow from data collection to fully automatic individualized and interpretable predictions. **a**, Video and touch data are recorded via the SenseToKnow application, which displays brief movies and a bubble-popping game (see Supplementary Video 1 for short clips of movies and Supplementary Video 2 showing a child playing the game). **b**, Faces are automatically detected using CVA, and the child’s face is identified and validated using sparse semi-automatic human annotations. Forty-nine facial landmarks, head pose and gaze coordinates are extracted for every frame using CVA. **c**, Automatic computation of multiple digital behavioral phenotypes. **d**, Training of the *K* = 1,000 XGBoost classifier from multiple phenotypes using fivefold cross-validation and overall performance evaluation, and estimation of the final prediction confidence score based on the Youden optimality index. **e**, Analysis of model interpretability using SHAP values analysis, showing features’ values in blue/red, and the direction of their contributions to the model prediction in blue/orange. **f**, An illustration (not real data) of how an individualized app administration summary report would provide information regarding a child’s unique digital phenotype (red dot on the graphs), along with group-wise distributions (ASD in orange and neurotypical in blue), confidence and quality scores and the app variables contributions to the individualized prediction.

The SenseToKnow app was administered during a pediatric primary care well-child visit to 475 (17–36 months old) toddlers, 49 of whom were subsequently diagnosed with autism and 98 of whom were diagnosed with DD–LD without autism (see Table 1 for demographic and clinical characteristics). The app elicited and quantified the child’s time attending to the screen, gaze to social versus nonsocial stimuli and to speech, facial dynamics complexity, frequency and complexity of head movements, response to name, blink rate and touch-based visual-motor behaviors. The app used ML to combine 23 digital phenotypes into the algorithm used for the diagnostic classification of the participants. Figure 1 illustrates the SenseToKnow app workflow from data collection to fully automatic individualized and interpretable diagnostic predictions.

**Table 1:** Study sample demographic and clinical characteristics

	Neurotypical (*n* = 328)	Autism (*n* = 49)	DD–LD (*n* = 98)
Age (in months)—mean (s.d.)	20.4 (3.0)	24.2 (4.6)	21.2 (3.55)
Sex (%)			
Boys	170 (51.8)	38 (77.5)	61 (62.0)
Girls	158 (48.2)	11 (22.5)	37 (38.0)
Ethnicity (%)			
Non-Hispanic/Latino	306 (93.3)	36 (73.4)	83 (84.7)
Hispanic/Latino	22 (6.7)	13 (26.6)	15 (15.3)
Race (%)			
Unknown/declined	0 (0.0)	0 (0.0)	1 (1.0)
American Indian/Alaskan Native	1 (0.3)	3 (6.1)	0 (0.0)
Asian	6 (1.8)	1 (2.0)	0 (0.0)
Black or African American	28 (8.5)	11 (22.4)	15 (15.3)
White/Caucasian	255 (77.7)	23 (46.9)	69 (70.4)
More than one race	32 (9.9)	7 (14.3)	8 (8.2)
Other	6 (1.8)	4 (8.3)	5 (5.1)
Highest level of education (%)			
Unknown/not reported	2 (0.6)	0 (0.0)	0 (0.0)
Without high school diploma	1 (0.3)	4 (8.2)	5 (5.1)
High school diploma or equivalent	12 (3.6)	8 (16.3)	8 (8.2)
Some college education	32 (9.8)	10 (20.4)	11 (11.2)
Four-year college degree or more	281 (85.7)	27 (55.1)	74 (75.5)
M-CHAT-R/F—total			
Unknown/not reported	1 (0.3)	2 (4.0)	0 (0.0)
Positive	2 (0.6)	38 (77.5)	18 (18.4)
Negative	325 (99.1)	9 (18.5)	80 (81.6)
ADOS calibrated severity score (CSS)			
Unknown/not reported—total (%)	N/A	6 (12.2)	85 (86.7)
Restricted/repetitive behavior CSS	N/A	7.76 (1.64)	5.23 (1.42)
Social affect CSS	N/A	6.97 (1.71)	3.77 (1.69)
Total CSS	N/A	7.41 (1.79)	3.69 (1.32)
Mullen Scales of Early Learning			
Unknown/not reported—total (%)	N/A	6 (12.2)	82 (100.0)
Early learning composite score	N/A	63.6 (10.12)	73.85 (15.30)
Expressive language *T*-score	N/A	28.34 (7.56)	35.23 (10.00)
Receptive language *T*-score	N/A	23.37 (5.60)	32.46 (12.94)
Fine motor *T* score	N/A	34.24 (10.06)	39.30 (6.60)
Visual reception *T* score	N/A	33.42 (10.60)	36.30 (12.03)

### Quality of app administration metrics

Quality scores were automatically computed for each app administration, which reflected the amount of available app variables weighted by their predictive power. In practice, these scores can be used to determine whether the app needs to be re-administered. Quality scores were found to be high (median score = 93.9%, Q1–Q3 (90.0–98.4%)), with no diagnostic group differences.

### Prediction confidence metrics

**Extended Data Fig. 1:**
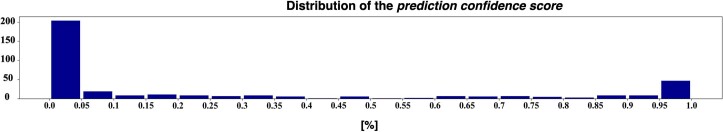
Distribution of the prediction confidence scores for the autistic and neurotypical groups. Participants having a prediction confidence score closer to 0 or 1 correspond to app variables either consistently related to neurotypical or autistic behavioral phenotypes.

A prediction confidence score for accurately classifying an individual child was also calculated. The heterogeneity of the autistic condition implies that some autistic toddlers will exhibit only a subset of the potential autism-related behavioral features. Similarly, nonautistic participants may exhibit behavioral patterns typically associated with autism (for example, display higher attention to nonsocial than social stimuli). The prediction confidence score quantified the confidence in the model’s prediction. As illustrated in Extended Data Fig. 1, the large majority of participants’ prediction confidence scores were rated with high confidence.

### Diagnostic accuracy of SenseToKnow for autism detection

**Fig. 2:**
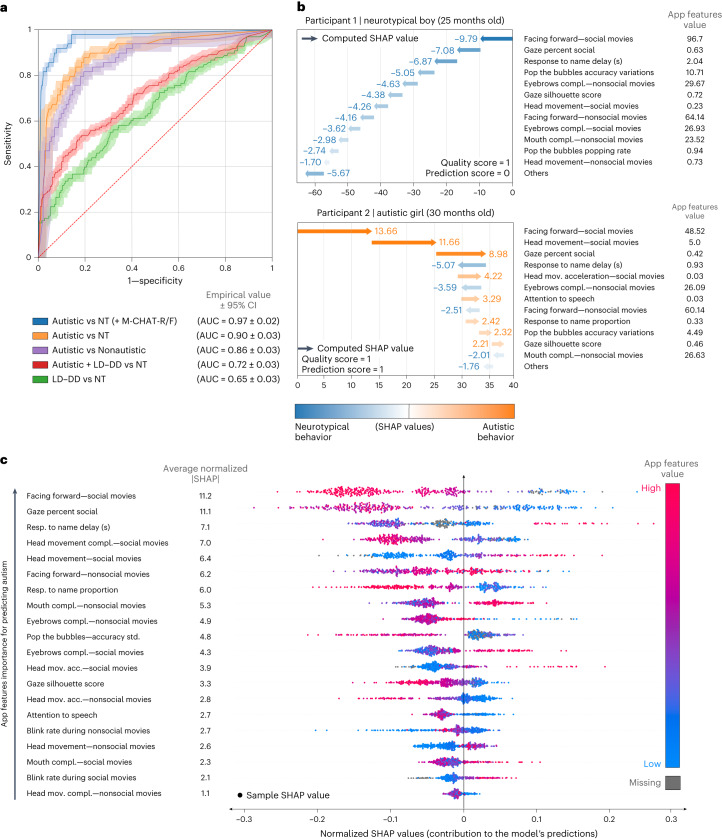
Accuracy metrics and normalized SHAP value analysis. **a**, ROC curve illustrating the performance of the model for classifying different diagnostic groups, using all app variables. *n* = 475 participants; 49 were diagnosed with autism and 98 were diagnosed with developmental delay or language delay without autism. The final score of the M-CHAT-R/F screening questionnaire was used when available (*n* = 374/377). Error bands correspond to 95% CI computed by the Hanley McNeil method. **b**, Examples of app administration reports are shown, one for a 25-month-old neurotypical boy and one for a 30-month-old autistic girl, both correctly classified, including each child’s app quality score, confidence score and the contributions of each app variable to the child’s individualized prediction. **c**, Normalized SHAP value analysis showing the app variables importance for the prediction of autism. The *x* axis represents the features’ contribution to the final prediction, with positive or negative values associated with an increase in the likelihood of an autism or neurotypical diagnosis, respectively. The *y* axis lists the app variables in descending order of importance. The blue–red color gradient indicates the relevance of each of the app variables to the score, from low to high values; gray indicates missing variables. For each app variable, a point represents the normalized SHAP value of an individual participant. NT, neurotypical.

Using all app variables, we trained a model comprised of *K* = 1,000 tree-based EXtreme Gradient Boosting (XGBoost) algorithms to classify diagnostic groups^26^. Figure 2a displays the area under the curve (AUC) results for the classification of autism versus each of the other groups (neurotypical, nonautism, developmental delay and/or language delay (DD–LD)), including accuracy based on the combination of the app results with the M-CHAT-R/F^2^, which was administered as part of the screening protocol.

Based on the Youden Index^27^, an algorithm integrating all app variables showed a high level of accuracy for the classification of autism versus neurotypical development with AUC = 0.90 (confidence interval (CI) (0.87–0.93)), sensitivity 87.8% (s.d. = 4.9) and specificity 80.8% (s.d. = 2.3). Restricting administrations to those with high prediction confidence, the AUC increased to 0.93 (CI (0.89–0.96)).

Classification of autism versus nonautism (DD–LD combined with neurotypical) also showed strong accuracy: AUC = 0.86 (CI (0.83–0.90)), sensitivity 81.6% (s.d. = 5.4) and specificity 80.5% (s.d. = 1.8). Table 2 shows performance results for autism versus neurotypical and autism versus nonautism (DD–LD and neurotypical combined) classification based on individual and combined app variables. Supplementary Table 1 provides the performances for all the cut-off thresholds defining the operating points of the associated receiver operating characteristic curve (ROC).

**Table 2:** App performance based on individual and combined app variables

	AUROC (95% CI)	Sensitivity	Specificity	PPV^a^	NPV^a^
All app variables	89.9 (3.0)	87.8 (4.9)	80.8 (2.3)	40.6 (8.8)	97.8 (99.7)
Facing forward	83.8 (3.7)	87.8 (4.4)	65.9 (2.6)	27.7 (5.2)	97.3 (99.6)
Gaze^b^	77.6 (4.0)	63.3 (7.7)	85.4 (1.8)	39.2 (8.4)	94.0 (99.1)
Facial dynamics complexity	75.9 (4.2)	63.3 (6.5)	82.9 (2.3)	35.6 (7.3)	93.8 (99.1)
Head movements	86.4 (3.4)	87.8 (4.1)	74.4 (2.4)	33.9 (6.8)	97.6 (99.7)
Response to name	65.8 (4.4)	83.7 (5.1)	46.6 (2.4)	19.0 (3.2)	95.0 (99.3)
Touch-based (game)	57.6 (4.5)	79.6 (5.2)	39.0 (2.5)	16.3 (2.7)	92.8 (8.9)
All app variables + M-CHAT-R/F score	96.6 (1.8)	91.8 (4.5)	92.1 (1.6)	63.4 (19.7)	98.7 (99.8)

Results represent the performance of the XGBoost model trained to classify autistic and neurotypical groups based on individual and combined app variables (digital phenotypes).

^a^PPV and NPV values adjusted for population prevalence (Supplementary Table 1).

^b^Gaze silhouette score, gaze speech correlation and gaze percent social.

AUROC, area under the ROC curve.

Nine autistic children who scored negative on the M-CHAT-R/F were correctly classified by the app as autistic, as determined by expert evaluation. Among 40 children screening positive on the M-CHAT-R/F, there were two classified neurotypical based on expert evaluation, and both were correctly classified by the app. Combining the app algorithm with the M-CHAT-R/F further increased classification performance to AUC = 0.97 (CI (0.96–0.98)), specificity = 91.8% (s.d. = 4.5) and sensitivity = 92.1% (s.d. = 1.6).

### Diagnostic accuracy of SenseToKnow for subgroups

Classification performance of the app based on AUCs remained largely consistent when stratifying groups by sex (AUC for girls = 89.1 (CI (82.6–95.6)), and for boys AUC = 89.6 (CI (86.2–93.0))), as well as race, ethnicity and age. Table 3 provides exhaustive performance results for all these subgroups, as well as the classification of autism versus DD–LD. However, CIs were larger due to smaller sample sizes for subgroups.

**Table 3:** App performance stratified by sex, race, ethnicity, age, quality score and prediction confidence threshold

	Group	*n*	NT	Correct	Not correct	AUC (%; 95% CI)	Sensitivity (STD)	Specificity (STD)	PPV (adjusted)	NPV (adjusted)
			Autism							
Sex	Boys	196	158	123	35	89.6 (3.4)	86.8 (5.3)	77.8 (3.2)	48.5 (7.7)	96.1 (99.6)
			38	33	5					
	Girls	181	170	142	28	89.1 (6.5)	90.9 (9.1)	83.5 (2.9)	26.3 (10.5)	99.3 (99.8)
			11	10	1					
Race	White	278	255	211	44	86.9 (4.9)	82.6 (7.8)	82.7 (2.4)	30.2 (9.2)	98.1 (99.5)
			23	19	4					
	Black	39	28	15	13	81.2 (8.5)	90.9 (9.0)	53.6 (9.5)	43.5 (4.0)	93.8 (99.6)
			11	10	1					
	Other	60	45	39	6	97.6 (2.8)	93.3 (7.2)	86.7 (4.6)	70.0 (12.9)	97.5 (99.8)
			15	14	1					
Ethnicity	Not Hispanic/Latino	342	306	245	61	87.8 (3.8)	86.1 (5.7)	80.1 (2.3)	33.7 (8.4)	98.0 (99.8)
			36	31	5					
	Hispanic/Latino	35	22	20	2	95.3 (4.3)	92.3 (7.1)	90.9 (6.2)	85.7 (17.7)	95.2 (99.8)
			13	12	1					
Age (months)	17–18.5	164	159	125	34	94.5 (7.1)	1.00 (0.0)	78.6 (2.8)	12.8 (9.0)	1.0 (1.0)
			5	5	0					
	18.5–24	104	86	72	14	89.5 (5.1)	83.3 (9.5)	83.7 (4.7)	51.7 (9.8)	96.0 (99.6)
			18	15	3					
	24–36	109	83	68	15	90.1 (4.2)	88.5 (6.0)	81.9 (4.3)	40.6 (8.8)	97.8 (99.7)
			26	23	3					
Quality score	Higher than 75%	349	310	259	51	89.6 (3.4)	84.6 (5.0)	83.5 (2.1)	39.3 (9.8)	97.7 (99.6)
			39	33	6					
	Lower than 75%	28	18	6	12	76.1 (10.0)	1.0 (0.0)	33.3 (12.3)	45.5 (3.1)	1.0 (1.0)
			10	10	0					
Prediction confidence threshold	Threshold 5%	251	216	201	15	92.6 (3.1)	91.4 (4.4)	93.1 (1.6)	68.1 (21.9)	98.5 (99.8)
			35	32	3					
	Threshold 10%	279	243	219	24	92.4 (3.0)	88.9 (4.9)	90.1 (2.1)	57.1 (16.0)	98.2 (99.7)
			36	32	4					
	Threshold 15%	297	258	228	30	92.0 (3.0)	89.7 (5.1)	88.4 (2.0)	53.8 (14.1)	98.3 (99.7)
			39	35	4					
	Threshold 20%	311	270	238	32	91.6 (3.0)	87.8 (5.4)	88.1 (1.7)	52.9 (13.6)	97.9 (99.7)
			41	36	5					
Diagnostic groups	Autistic versus nonautistic	475	426^a^	343	83	86.4 (3.4)	81.6 (5.4)	80.5 (1.8)	32.5 (8.2)	97.4 (99.5)
			49^b^	40	9					
	Autistic + DD–LD versus NT	475	328^c^	267	61	71.7 (2.7)	53.7 (3.9)	81.4 (2.1)	56.4 (5.8)	79.7 (98.8)
			147^d^	79	68					
	DD–LD versus NT	426	328^c^	227	101	65.1 (3.3)	55.1 (5.2)	69.2 (2.6)	34.8 (3.7)	83.8 (98.6)
			98^e^	54	44					
	Autistic versus DD–LD	426	49^b^	10	39	83.3 (3.9)	80.1 (6.0)	74.6 (4.3)	60.9 (6.2)	88.0 (99.4)
			98^e^	73	25					

The operating point (or positivity threshold) corresponds to the one maximizing the Youden index. PPV and NPV values were adjusted for population prevalence. Stratification by diagnosis group refers to neurotypical (NT; first row) and autistic (second row) except for the diagnostic groups category;

^a^Nonautistic group (neurotypical + DD–LD).

^b^Autistic.

^c^Neurotypical (NT).

^d^Autistic + DD–LD.

^e^DD–LD.

Correct, number of correct diagnosis predictions; not correct, number of incorrect predictions.

### Model interpretability

**Fig. 3:**
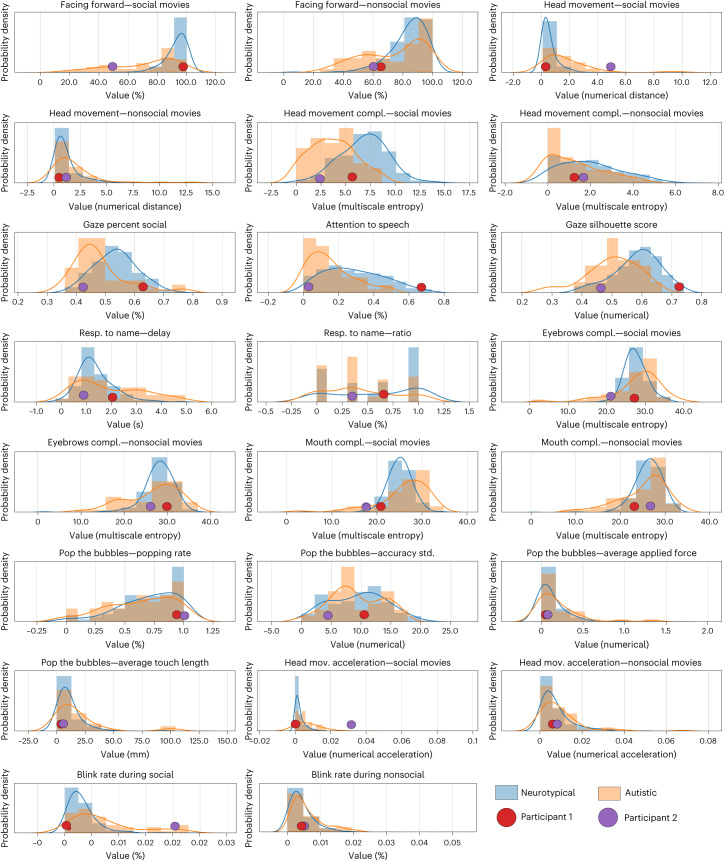
Distributions for each of the app variables. Empirical probability distributions of all nonmissing samples of the app variables are shown for all autistic (*n* = 49, orange) and neurotypical (*n* = 328, blue) participants. The app variables values for one neurotypical (red) and one autistic (purple) participant who were correctly classified are overlayed on the distributions.

Distributions for each app variable for autistic and neurotypical participants are shown in Fig. 3. To address model interpretability, we used SHapley Additive exPlanations (SHAP) values^28^ for each child to examine the relative contributions of the app variables to the model’s prediction and disambiguate the contribution of each feature from their missingness (Fig. 2b,c). Figure 2c illustrates the ordered normalized importance of the app variables for the overall model. Facing forward during social movies was the strongest predictor (mean |SHAP| = 11.2% (s.d. = 6.0%)), followed by the percent of time gazing at social stimuli (mean |SHAP| = 11.1% (s.d. = 5.7%)) and delay in response to a name call (mean |SHAP| = 7.1% (s.d. = 4.9%)). The SHAP values as a function of the app variable values are provided in Supplementary Fig. 1.

**Extended Data Fig. 2:**
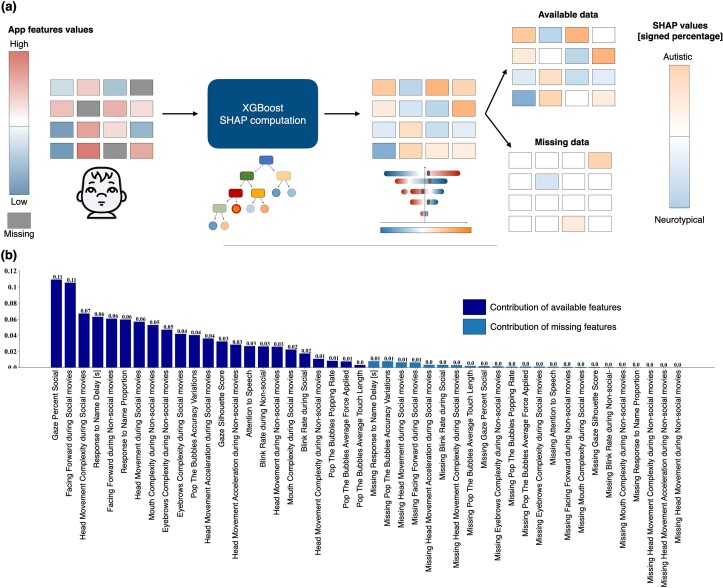
Present and missing app variables’ contributions to the predictions. Illustration of the computation of the variables contributions for present and missing app variables (**a**), and normalized variables contribution for discriminating autistic from neurotypical participants, including the contribution of missing variables (**b**). Note that only the contributions of available variables (in dark blue) are used to compute the variables importance used in the computation of the quality score.

SHAP interaction values indicated that interactions between predictors were substantial contributors to the model; average contribution of app variables alone was 64.6% (s.d. = 3.4%) and 35.4% (s.d. = 3.4%) for the feature interactions. Analysis of the missing data SHAP values revealed that missing variables were contributing to 5.2% (s.d. = 13.2%) of the model predictions, as illustrated in Extended Data Fig. 2.

### Individualized interpretability

**Extended Data Fig. 3:**
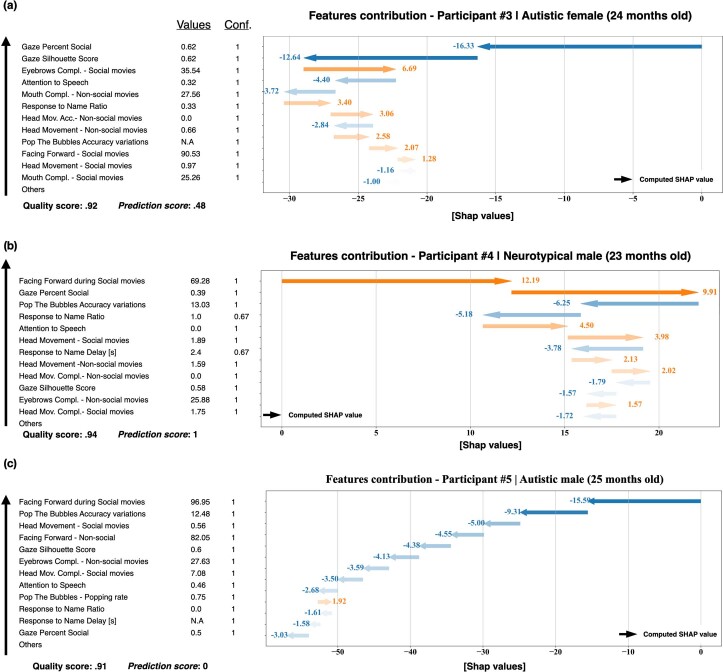
Additional illustrative digital phenotypes. (**a**) An autistic girl who did not receive the M-CHAT-R/F. Her digital phenotype shows a mix of autistic and neurotypical-related variables, as illustrated in her SHAP values and prediction confidence score of .48. (**b**) App variables contributions of a misclassified neurotypical participant, whose digital phenotype was typically associated with autistic behavioral patterns. (**c**) App variables of a misclassified autistic participant, whose digital phenotype was typically associated with neurotypical patterns. Note that even misclassifications are provided with detailed explanations by the proposed framework. SHAP values of these participants are reported in Supplementary Fig. 1 of the Supplementary Information with gray, green and sky-blue points.

Analysis of the individual SHAP values revealed individual behavioral patterns that explained the model’s prediction for each participant. Figure 2b shows individual cases illustrating how the positive or negative contributions of the app variables to the predictions can be used to (1) deliver intelligible explanations about the child’s app administration and diagnostic prediction, (2) highlight individualized behavioral patterns associated with autism or neurotypical development and (3) identify misclassified digital profile patterns. Extended Data Fig. 3 shows the following three additional illustrative cases: participant 3—an autistic child who did not receive an M-CHAT-R/F administration; participant 4—a neurotypical child incorrectly predicted as autistic; and participant 5—an autistic participant incorrectly predicted as neurotypical. The framework also enables us to provide explanations for the misclassified cases.

## Discussion

When used in primary care, the accuracy of autism screening parent questionnaires has been found to be lower than in research contexts, especially for children of color and girls, which can increase disparities in access to early diagnosis and intervention. Studies using eye-tracking of social attention alone as an autism screening tool have reported inadequate sensitivity, perhaps because assessments based on only one autism feature (differences in social attention) do not adequately capture the complex and heterogeneous clinical presentation of autism^13^.

We evaluated the accuracy of an ML and CVA-based algorithm using multiple autism-related digital phenotypes assessed via a mobile app (SenseToKnow) administered on a tablet in pediatric primary care settings for identification of autism in a large sample of toddler-age children, the age at which screening is routinely conducted. The app captured the wide range of early signs associated with autism, including differences in social attention, facial expressions, head movements, response to name, blink rates and motor skills, and was robust to missing data. ML allowed optimization of the prediction algorithm based on weighting different behavioral variables and their interactions. We demonstrated high levels of usability of the app based on quality scores that were automatically computed for each app administration based on the amount of available app variables weighted by their predictive power.

The screening app demonstrated high diagnostic accuracy for the classification of autistic versus neurotypical children with AUC = 0.90, sensitivity = 87.8%, specificity = 80.8%, negative predictive value (NPV) = 97.8% and PPV = 40.6%, with similar sensitivity levels across sex, race and ethnicity. Diagnostic accuracy for the classification of autism versus nonautism (combining neurotypical and DD–LD groups) was similarly high. The fact that the sensitivity of SenseToKnow for detecting autism did not differ based on the child’s sex, race or ethnicity suggests that an objective digital screening approach that relies on direct quantitative observations of multiple behaviors may improve autism screening in diverse populations. Specificity levels for boys versus girls and for Hispanic/Latino versus non-Hispanic/Latino children were similar, whereas specificity was lower for Black children (53.6%) compared to White (82.7%) and other races (86.7%). There is a clear need for further research with larger samples to more fully assess the app’s performance based on race, ethnicity, sex and age differences. Such studies are underway.

We developed methods for automatic assessment of the quality of the app administration and prediction confidence scores, both of which could facilitate the use of SenseToKnow in real-world settings. The quality score provides a simple, actionable means of determining whether the app should be re-administered. This can be combined with a prediction confidence score, which can inform a provider about the degree of certainty regarding the likelihood a child will be diagnosed with autism. Children with uncertain values could be followed to determine whether autism signs become more pronounced, whereas children with high confidence values could be prioritized for referral or begin intervention while the parent waits for their child to be evaluated. Using SHAP analyses, the app output provides interpretable information regarding which behavioral features are contributing to the diagnostic prediction for an individual child. Such information could be used prescriptively to identify areas in which behavioral intervention should be targeted. This approach is supported by a recent study that included some participants in the present sample that examined the concurrent validity of the individual digital phenotypes generated by the app and reported significant correlations between specific digital phenotypes and several independent, standardized measures of autism-related behaviors, as well as social, language, cognitive and motor abilities^29^. Notably, the app quantifies autism signs related to social attention, facial expressions, response to language cues and motor skills, but does not capture behaviors in the restricted and repetitive behavior domain.

In the context of an overall pathway for autism diagnosis, our vision is that autism screening in primary care should be based on integrating multiple sources of information, including screening questionnaires based on parent report and digital screening based on direct behavioral observation. Recent work suggests that ML analysis of a child’s healthcare utilization patterns using data passively derived from the electronic health record (EHR) could also be useful for early autism prediction^30^. Results of the present study support this multimodal screening approach. A large study conducted in primary care found that the PPV of the M-CHAT/F was 14.6% and was lower for girls and children of color^6^. In comparison, the PPV of the app in the present study was 40.6%, and the app performed similarly across children of different sex, race and ethnicity. Furthermore, combining the M-CHAT-R/F with digital screening resulted in an increased PPV of 63.4%. Thus, our results suggest that a digital phenotyping approach will improve the accuracy of autism screening in real-world settings.

Limitations of the present study include possible validation bias given that it was not feasible to conduct a comprehensive diagnostic evaluation on participants considered neurotypical. This was mitigated by the fact that diagnosticians were naïve with respect to the app results. The percentage of autism versus nonautism cases in this study is higher than in the general population, raising the potential for sampling bias. It is possible that parents who had developmental concerns about their child were more likely to enroll the child in the study. Although prevalence bias is addressed statistically by calibrating the performance metrics to the population prevalence of autism, this remains a limitation of the study. Accuracy assessments potentially could have been inflated due to differences in language abilities between the autism and DD groups, although the two groups had similar nonverbal abilities. Future studies are needed to evaluate the app’s performance in an independent sample with children of different ages and language and cognitive abilities. This study has several strengths, including its diverse sample, the evaluation of the app in a real-world setting during the typical age range for autism screening, and the follow-up of children up to the age of 4 years to determine their final diagnosis.

We conclude that quantitative, objective and scalable digital phenotyping offers promise in increasing the accuracy of autism screening and reducing disparities in access to diagnosis and intervention, complementing existing autism screening questionnaires. Although we believe that this study represents a substantial step forward in developing improved autism screening tools, accurate use of these screening tools requires training and systematic implementation by primary providers, and a positive screen must then be linked to appropriate referrals and services. Each of these touch points along the clinical care pathway contributes to the quality of early autism identification and can impact timely access to interventions and services that can influence long-term outcomes.

## Methods

### Study cohort

The study was conducted from December 2018 to March 2020 (Pro00085434). Participants were 475 children, 17–36 months, who were consecutively enrolled at one of four Duke University Health System (DUHS) pediatric primary care clinics during their well-child visit. Inclusion criteria were age 16–38 months, not ill and caregiver’s language was English or Spanish. Exclusion criteria were sensory or motor impairment that precluded sitting or viewing the app, unavailable clinical data and child too upset at their well-child visit^29^. Table 1 describes sample demographic and clinical characteristics.

In total, 754 participants were approached and invited to participate, 214 declined participation and 475 (93% of enrolled participants) completed study measures. All parents or legal guardians provided written informed consent, and the study protocol (Pro00085434) was approved by the DUHS Institutional Review Board.

### Diagnostic classification

Children were administered the M-CHAT-R/F^2^, a parent survey querying different autism signs. Children with a final M-CHAT-R/F score of >2 or whose parents and/or provider expressed any developmental concern were provided a gold standard autism diagnostic evaluation based on the Autism Diagnostic Observation Schedule-Second Edition (ADOS-2)^31^, a checklist of ASD diagnostic criteria based on the American Psychiatric Association Diagnostic and Statistical Manual of Mental Disorders, Fifth Edition (DSM-5), and Mullen Scales of Early Learning^32^, which was conducted by a licensed, research-reliable psychologist who was naïve with respect to app results^29^. Mean length of time between app screening and evaluation was 3.5 months, which is a similar or shorter duration compared to real-world settings. Diagnosis of ASD required meeting full DSM-5 diagnostic criteria. Diagnosis of DD–LD without autism was defined as failing the M-CHAT-R/F and/or having provider or parent concerns, having been administered the ADOS-2 and Mullen scales and determined by the psychologist not to meet diagnostic criteria for autism, and exhibiting DD–LD based on the Mullen scales (scoring ≥9 points below the mean on at least one Mullen scales subscale; s.d. = 10).

In addition, each participant’s DUHS EHR was monitored through age 4 years to confirm whether the child subsequently received a diagnosis of either ASD or DD–LD. Following validated methods used in ref. ^6^, children were classified as autistic or DD–LD based on their EHR record if an International Classification of Diseases, Ninth and Tenth Revisions diagnostic code for ASD or DD–LD (without autism) appeared more than once or was provided by an autism specialty clinic. If a child did not have an elevated M-CHAT-R/F score, no developmental concerns were raised by the provider or parents, and there were no autism or DD–LD diagnostic codes in the EHR through age 4 years, they were considered neurotypical. There were two children classified as neurotypical who scored positive on the M-CHAT-R/F and were considered neurotypical based on expert diagnostic evaluation and had no autism or DD–LD EHR diagnostic codes.

Based on these procedures, 49 children were diagnosed with ASD (six based on EHR only), 98 children were diagnosed with DD–LD without autism (78 based on EHR only) and 328 children were considered neurotypical. Diagnosis of autism or DD was made naïve to app results.

### SenseToKnow app stimuli

**Extended Data Fig. 4:**
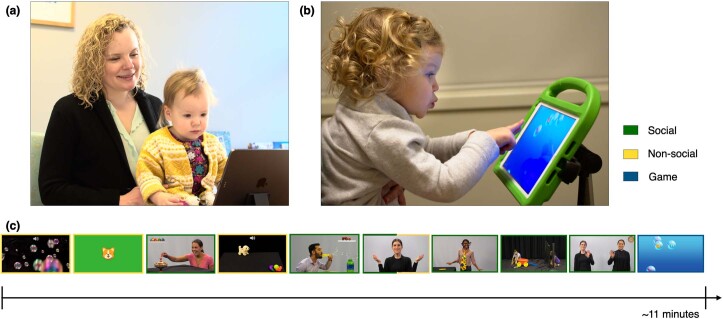
SenseToKnow app administration and movies. (**a**) An illustrative example of the app administration, a toddler watches a set of developmentally appropriate movies on a tablet (see Video 1 online). (**b**) After watching the movies, participants play a ‘bubble popping’ game (see Video 2 online). (**c**) Illustration of the movies presented (in order), from left to right. The movies are referred to as: Floating Bubbles, Dog in Grass, Spinning Top, Mechanical Puppy, Blowing Bubbles, Rhymes and Toys, Make Me Laugh, Playing with Blocks, and Fun at the Park. Around each image representing the movies, a green/yellow box indicates if the movies present mainly social or non-social content. Movies are presented in English or Spanish and include actors of diverse ethnic/racial backgrounds.

The parent held their child on their lap while brief, engaging movies were presented on an iPad set on a tripod approximately 60 cm away from the child. The parent was asked to refrain from talking during the movies. The frontal camera embedded in the device recorded the child’s behavior at resolutions of 1280 × 720, 30 frames per second. While the child was watching the movies, their name was called three times by an examiner standing behind them at predefined timestamps. The child then participated in a bubble-popping game using their fingers to pop a set of colored bubbles that moved continuously across the screen. App completion took approximately 10 min. English and Spanish versions were shown^29^. The stimuli (brief movies) and game used in the app are illustrated in Fig. 1, Extended Data Fig. 4 and Supplementary Videos 1 and 2. Consent was obtained from all individuals (or their parents or guardians) whose faces are shown in the figures or videos for publication of these images.

### Description of app variables

CVA was used for the identification and recognition of the child’s face and the estimation of the frame-wise facial landmarks, head pose and gaze^19^. Several CVA-based and touch-based behavioral variables were computed, described next^29^.

#### Facing forward

During the social and nonsocial movies (Supplementary Video 1), we computed the average percentage of time the children faced the screen, filtering in frames using the following three rules: eyes were open, estimated gaze was at or close to the screen area and the face was relatively steady, referred to as facing forward. This variable was used as a proxy for the child’s attention to the movies^19^.

#### Social attention

The app includes two movies featuring clearly separable social and nonsocial stimuli on each side of the screen designed to assess the child’s social/nonsocial attentional preference (Supplementary Video 1). The variable gaze percent social was defined as the percentage of time the child gazed at the social half of the screen, and the gaze silhouette score reflected the degree to which the gaze clusters concentrated on specific elements of the video (for example, person or toy) versus spread out^19^.

#### Attention to speech

One of the movies features two actors, one on each side of the screen, taking turns in a conversation (Supplementary Video 1). We computed the correlation between the child’s gaze patterns and the alternating conversation, defined as the gaze speech correlation variable^19^.

#### Facial dynamics complexity

The complexity of the facial landmarks’ dynamics was estimated for the eyebrows and mouth regions of the child’s face using multiscale entropy. We computed the average complexity of the mouth and eyebrows regions during social and nonsocial movies, referred to as the mouth complexity and eyebrows complexity^20^.

#### Head movement

We evaluated the rate of head movement (computed from the time series of the facial landmarks) for social and nonsocial movies (Supplementary Video 1). Average head movement was referred to as head movement. Complexity and acceleration of the head movements were computed for both types of stimuli using multiscale entropy and the derivative of the time series, respectively^22^.

#### Response to name

Based on automatic detection of the name calls and the child’s response to their name by turning their head computed from the facial landmarks, we defined the following two CVA-based variables: response to name proportion, representing the proportion of times the child oriented to the name call, and response to name delay, the average delay (in seconds) between the offset of the name call and head turn^23^.

#### Blink rate

During the social and nonsocial movies, CVA was used to extract the blink rates as indices of attentional engagement, referred to as blink rate^24^.

#### Touch-based visual-motor skills

Using the touch and device kinetic information provided by the device sensors when the child played the bubble-popping game (Supplementary Video 2), we defined touch popping rate as the ratio of popped bubbles over the number of touches, touch error s.d. as the standard deviation of the distance between the child’s finger position when touching the screen and the center of the closest bubble, touch average length as the average length of the child’s finger trajectory on the screen and touch average applied force as the average estimated force applied on the screen when touching it^25^.

**Extended Data Fig. 5:**
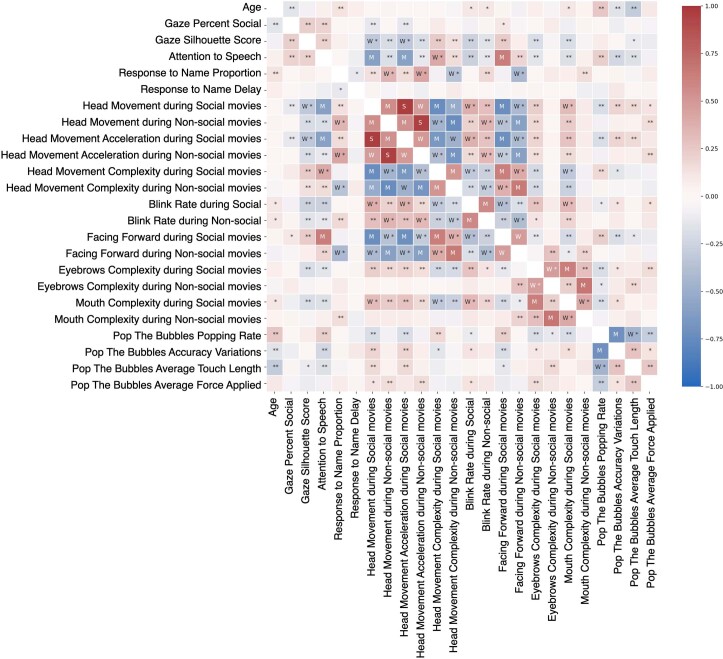
App variables pairwise correlation coefficients. ‘W,’ ‘M,’ and ‘S’ denote Weak, Medium, and Strong associations, respectively. An association between two variables was considered weak if their Spearman rho correlation coefficient was higher than 0.3 in absolute value, 0.5 for a medium association, and 0.5 for a strong association. We used a two-sided Spearman’s rank correlation test to test. No adjustment for multiple comparisons were made. *: p-value < 0.05; **: p-value < 0.01; ***: p-value < 0.001.

**Extended Data Fig. 6:**
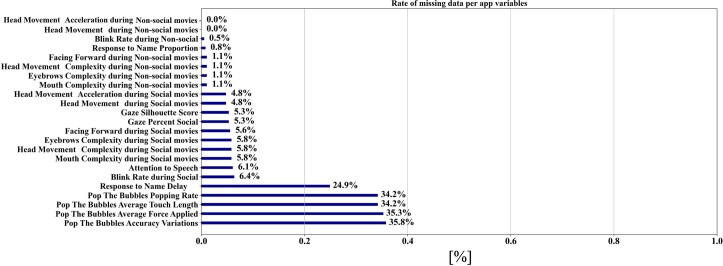
Rate of missing data per app variables. For each variable, we computed the number of missing data over the sample size. As we can observe, the rate of missingness is relatively low, with a higher percentage in the case of the average delay when responding to the name calls. This is expected since participants who did not respond to the name calls miss this variable.

In total, we measured 23 app-derived variables, comprising 19 CVA-based and four touch-based variables. The app variables pairwise correlation coefficients and the rate of missing data are shown in Extended Data Figs. 5 and 6, respectively.

### Statistical analyses

Using the app variables, we trained a model comprising *K* = 1,000 tree-based XGBoost algorithms to differentiate diagnostic groups^26^. For each XGBoost model, fivefold cross-validation was used while shuffling the data to compute individual intermediary binary predictions and SHAP value statistics (metrics mean and s.d.)^28^. The final prediction confidence scores, between 0 and 1, were computed by averaging the *K* predictions. We implemented a fivefold nested cross-validation stratified by diagnosis group to separate the data used for training the algorithm and the evaluation of unseen data^33^. Missing data were encoded with a value out of the range of the app variables, such that the optimization of the decision trees considered the missing data as information. Overfitting was controlled using a tree maximum depth of 3, subsampling app variables at a rate of 80% and using regularization parameters during the optimization process. Diagnostic group imbalance was addressed by weighting training instances by the imbalance ratio. Details regarding the algorithm and hyperparameters are provided below. The contribution of the app variables to individual predictions was assessed by the SHAP values, computed for each child using all other data to train the model and normalized such that the features’ contributions to the individual predictions range from 0 to 1. A quality score was computed based on the amount of available app variables weighted by their predictive power (measured as their relative importance to the model).

Performance was evaluated using the ROC AUC, with 95% CIs computed using the Hanley McNeil method^34^. Unless otherwise mentioned, sensitivity, specificity, PPV and NPV were defined using the operating point of the ROC that optimized the Youden index, with an equal weight given to sensitivity and specificity^27^. Given that the study sample autism prevalence (${\pi }_{{\mathrm{study}}}=\frac{49}{328}\approx 14.9 \%$) differs from the general population in which the screening tool would be used (*π*_population_ ≈ 2%), we also report the adjusted PPV and NPV to provide a more accurate estimation of the app performance as a screening tool deployed at scale in practice. Statistics were calculated in Python V.3.8.10, using SciPy low-level functions V.1.7.3, XGBoost and SHAP official implementations V.1.5.2 and V.0.40.0, respectively.

### Computation of the prediction confidence score

The prediction confidence score was used to compute the model performance and assess the certainty of the diagnostic classification prediction. Given that autism is a heterogeneous condition, it is anticipated that some autistic children will only display a subset of potential autism signs. Similarly, it is anticipated that neurotypical children will sometimes exhibit behaviors typically associated with autism. From a data science perspective, these challenging cases may be represented in ambiguous regions of the app variables space, as their variables might have a mix of autistic and neurotypical-related values. Therefore, the decision boundaries associated with these regions of the variable space may fluctuate when training the algorithm over different splits of the dataset, which we used to reveal the difficult cases. We counted the proportion of positive and negative predictions of each participant, over the *K* = 1,000 experiments. The distribution of the averaged prediction for each participant (which we called the prediction confidence score; Extended Data Fig. 1) shows participants with consistent neurotypical predictions (prediction confidence score close to 0; at the extreme left of Extended Data Fig. 1) and with consistent autistic predictions (prediction confidence score close to 1; at the extreme right of Extended Data Fig. 1). The cases in between are considered more difficult because their prediction fluctuated between the two groups over the different training of the algorithm. We considered conclusive the administrations whose predictions were the same in at least 80% of the cases (either positive or negative predictions) and inconclusive otherwise. Interestingly, as illustrated in Extended Data Fig. 1, the prediction confidence score can be related to the SHAP values of the participants. Indeed, conclusive administrations of the app have app variables contributions to the prediction that point to the same direction (either toward a positive or negative prediction), while inconclusive administrations show a mix of positive and negative contributions of the app variables.

### XGBoost algorithm implementation

**Extended Data Fig. 7:**
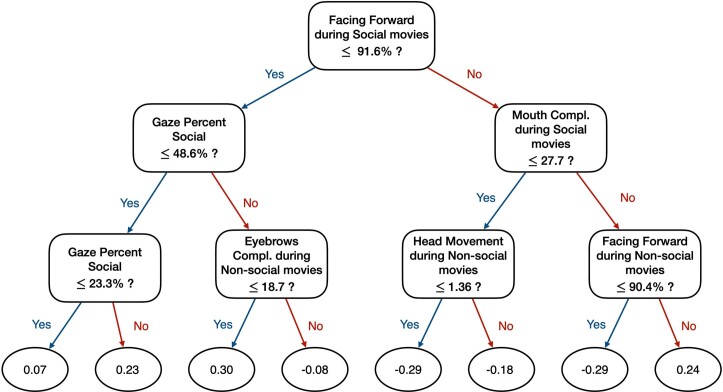
Sample of one of the XGBoost optimized trees. The final leaf score attributed to a participant on this tree depends on the value of their app variables. The final prediction is computed averaging the leaf scores of the 100 estimators.

XGBoost algorithm is a popular model based on several decision trees whose node variables and split decisions are optimized using gradient statistics of a loss function. It constructs multiple graphs that examine the app variables under various sequential ‘if’ statements. The algorithm progressively adds more ‘if’ conditions to the decision tree to improve the predictions of the overall model. We used the standard implementation of XGBoost as provided by the authors^26^. We used all default parameters of the algorithms, except the ones in bold that we changed to account for the relatively small sample size and the class imbalance, and to prevent overfitting. n_estimators = 100; **max_depth** = **3** (default is 6, prompt to overfitting in this setting); objective = ‘binary:logistic’; booster = ‘gbtree’; **tree_method** = **‘exact’** instead of ‘auto’ because the sample size is relatively small; **colsample_bytree** = **0.8** instead of 0.5 due to the relatively small sample size; subsample = 1; **colsumbsample** = **0.8** instead of 0.5 due to the relatively small sample size; **learning_rate** = **0.15** instead of 0.3; **gamma** = **0.1** instead of 0 to prevent overfitting, as this is a regularization parameter; reg_lambda = 0.1; alpha = 0. Extended Data Fig. 7 illustrates one of the estimators of the trained model.

### SHAP computation

The SHAP values measure the contribution of the app variables to the final prediction. They measure the impact of having a certain value for a given variable in comparison to the prediction we would be making if that variable took a baseline value. Originating in the cooperative game theory field, this state-of-the-art method is used to shed light on ‘black box’ ML algorithms. This framework benefits from strong theoretical guarantees to explain the contribution of each input variable to the final prediction, accounting and estimating the contributions of the variable’s interactions.

In this work, the SHAP values were computed and stored for each sample of the test sets when performing cross-validation, that is, training a different model every time with the rest of the data. Therefore, we needed to normalize the SHAP values first to compare them across different splits. The normalized contribution of the app variable was denoted as ${k}(k\in \left[1,{K}\right])$, for an individual ${i}(i\in \left[1,{n}\right])$, is ${\phi }_{k,{\mathrm{normalized}}}^{i}=\frac{{\phi }_{k}^{i}}{\mathop{\sum }\nolimits_{k=1}^{K}{|\phi }_{k}^{i}|}\in [-1,1]$. We conserved the sign of the SHAP values as it indicates the direction of the contribution, either toward autistic or neurotypical-related behavioral patterns.

In the learning algorithm used, being robust to missing values, an individual $i$ may have a missing value for variable $k$, which will be used by the algorithm to compute a diagnosis prediction. In this case, the contribution (that is, a SHAP value) of the missing data to the final prediction, still denoted as ${\phi }_{k}^{i}$, accounts for the contribution of this variable being missing.

To disambiguate the contribution of actual variables from their missingness, we set to 0 the SHAP value associated with variable $k$ for that sample and defined as ${\phi }_{{Z}_{k}}^{i}$ the contribution of having variable $k$ missing for that sample. This is illustrated in Extended Data Fig. 2.

This process leads to $2{NK}$ SHAP values for the study cohort, used to compute:

- The importance of variable $k$ to the model as the average contribution of that variable is measured as ${{{\phi }}}_{{\rm{k}}}=\frac{1}{n}\mathop{\sum }\nolimits_{i=1}^{n}|{\phi }_{k}^{i}|\in \left[0,1\right]$. These contributions are represented in dark blue in Extended Data Fig. 2b.
- The importance of the missingness of variable $k$ to the model, measured as the average contribution of the missingness of that variable as follows: ${{{\phi }}}_{{Z}_{k}}=\frac{1}{n}\mathop{\sum }\nolimits_{i=1}^{n}|{\phi }_{{Z}_{k}}^{i}|\in \left[0,1\right]$. These contributions are represented in sky blue in Extended Data Fig. 2b.

### Computation of the app variables confidence score

Given the set of app variables ${\left({x}_{k}^{\,i}\right)}_{k\in [1,K]}$ for a participant *i*, we first compute a measure of confidence (or certainty) of each app variable, denoted by ${\left({\rho }_{k}^{i}\right)}_{k\in [1,K]}$. The intuition behind the computation of these confidence scores follows the weak law of large numbers, which states that the average of a sufficiently large number of observations will be close to the expected value of the measure. We describe next the computation of the app variables confidence scores $\rho$.

**Extended Data Fig. 8:**
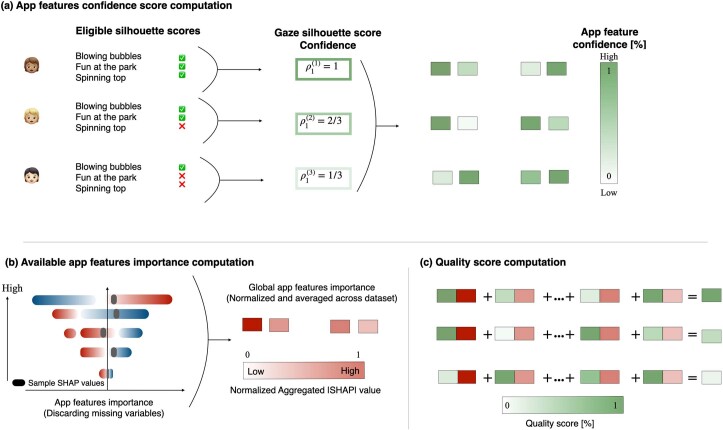
Illustration of the different steps to compute the quality score. (**a**) Computation of the confidence score for each app variable. This score accounts for how many times the measurement was available and resulted in a confidence score between 0 and 1. (**b**) Computation of the app variables importance. These scores are normalized and represent the average contribution of each app variable to the model performances. See Fig. 2-c where actual numbers are reported. Note that (i) these scores are global (as computed from all participants’ SHAP values) and fixed to compute the quality score of all participants and (ii) missing data were discarded following the methodology explained in Extended Data Fig. 2 to estimate the true importance of each app variable when they were available. (**c**) Computation of the quality score as a weighted sum of the confidence score by the variables importance.

- As illustrated in Extended Data Fig. 8, some app variables are computed as aggregates of several measurements. For instance, the gaze percent social variable is the average percentage the participants spent looking at the social part of two of the presented movies. The confidence ${\rho }_{k}^{i}$ of an aggregated variable $k$ for participant *i* is the ratio of available measurements computed for participant *i* over the maximum number of measurements to compute that variable. Reasons for missing a variable for a movie include (1) the child did not attend to enough of the movie to trust the computation of that measurement, (2) the movie was not presented to the participant due to technical issues or (3) the administration of the app was interrupted.
- For the two variables related to the participant’s response when their name is called, namely the proportion of response and the average delay when responding, the confidence score was the proportion of valid name-call experiments. Because their name was called a maximum of three times, the confidence score ranges from 0/3 to 3/3.
- For the variables collected during the bubble-popping game, we used as a measure of confidence the number of times the participant touched the screen. The confidence score is proportional to the number of touches when it is below or equal to 15, with 1 for higher number of touches and 0 otherwise.
- The confidence score of a missing variable is set to 0.

### Computation of the app variables predictive power

When assessing the quality of the administration, one might want to put more weight on variables that contribute the most to the predictive performances of the model. Therefore, to compute the quality score of an administration, we used the normalized app variables importance ${(G\left({X}_{k}\right))}_{k\in [1,{K}]}\,$ to weight the app variables. Note that for computing the predictive power of the app variables, we used only the SHAP values of available variables, setting to 0 the SHAP values of missing variables.

### Computation of the app administration quality score

A quality score is computed for each app administration, based on the amount of available information computed using the app data and weighted by the predictive ability (or variables importance) of each of the app variables. This score, between 0 and 1, quantifies the potential for the collected data on the participant to lead to a meaningful prediction of autism.

After we compute for each administration *i* the confidence score ${\left({\rho }_{k}^{i}\right)}_{k\in [1,K]}$ of each app variable ${\left({x}_{k}^{\,i}\right)}_{k\in [1,K]}$ and gain an idea of their expected predictive power ${({E}_{X}[G\left({X}_{k}\right)])}_{k\in [1,{K}]}$, the quality score is computed as

$${\rm{Quality}}\; {\rm{score}}\left({x}^{\,{\rm{i}}}\right)=\mathop{\sum }\limits_{k=0}^{K}{E}_{X}\left[G\left({X}_{k}\right)\right]\,{\rho }_{k}^{i}.$$

**Extended Data Fig. 9:**
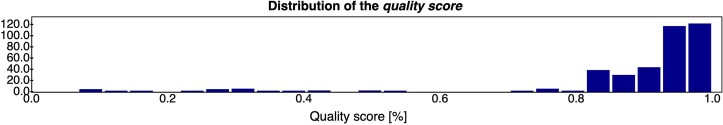
Distribution of the quality score of the analyzed cohort. A quality score close to 1 implies an administration with all app variables computed, while a quality score close to 0 implies that none of the app variables were collected during the assessment.

When all variables are missing, ${\left({\rho }_{k}^{i}\right)}_{k\in [1,{K}]}=(0,\ldots ,0)$, the score is equal to 0, and when all the app variables are measured with the maximum amount of information, ${\left({\rho }_{k}^{i}\right)}_{k\in [1,{K}]}=(1,\ldots ,1)$, then the quality score is equal to the sum of normalized variables contributions, which is equal to 1. Extended Data Fig. 9 shows the distribution of the quality score.

### Adjusted/calibrated PPV and NPV scores

The prevalence of autism in the cohort analyzed in this study, as in many studies in the field, differs from the reported prevalence of autism in the broader population. While the 2018 prevalence of autism in the United States is of 1 over 44 (${{{\pi }}}_{{\rm{population}}}=\frac{1}{44}\approx 2.3 \%$), the analyzed cohort in this study is composed of 49 autistic participants and 328 nonautistic participants (${{{\pi }}}_{{\rm{population}}}=\frac{49}{328}\approx 14.9 \%$). Some screening tool performance metrics, such as the specificity, sensitivity or the area under the ROC curve, are invariant to such prevalence differences, as their values do not depend on the group ratio (for example, the sensitivity only depends on the measurement tool performance on the autistic group; the specificity only depends on the measurement tool performance on the nonautistic group). Therefore, providing an unbiased sampling of the population and a large enough sample size, the reported prevalence-invariant metrics should provide a good estimate of what would be the value of those metrics if the tool were implemented in the general population.

However, precision-based performance measures, such as the precision (or PPV), the NPV or the ${{\rm{F}}}_{{{\beta }}}$ scores depend on the autism prevalence in the analyzed cohort. Thus, these measures provide inaccurate estimates of the expected performance when the measurement tool is deployed outside of research settings.

Therefore, we now report the expected performance we would have if the autism prevalence in this study was the same as that in the general population, following the procedure detailed in Siblini et al.^35^

For a reference prevalence, ${\pi }_{{\mathrm{population}}}$, and a study prevalence of$\,{\pi }_{{\mathrm{study}}}$, the corrected PPV (or precision), corrected NPV and ${F}_{{{\beta }}}$ are:

$$\begin{array}{l}{{\mathrm{PPV}}}_{C}=\frac{{\rm{TP}}}{{\rm{TP}}+\frac{{{{\pi }}}_{{\rm{study}}}\left(1-{{{\pi }}}_{{\rm{population}}}\right)}{{{{\pi }}}_{{\rm{population}}}\left(1-{{{\pi }}}_{{\rm{study}}}\right)}{\rm{FP}}}, \\ {F}_{\beta ,{C}}=\left(1+{{{\beta }}}^{2}\right)\frac{{{\mathrm{Precision}}}_{C}.{\mathrm{Sensitivity}}}{{{{\beta }}}^{2}{\mathrm{Sensitivity}}+{{\mathrm{Precision}}}_{C}}, \\ {\mathrm{and}}\;{{\mathrm{NPV}}}_{C}=\frac{\frac{{{{\pi }}}_{{\rm{study}}}\left(1-{{{\pi }}}_{{\rm{population}}}\right)}{{{{\pi }}}_{{\rm{population}}}\left(1-{{{\pi }}}_{{\rm{study}}}\right)}{\rm{TN}}}{{\rm{FN}}+\frac{{{{\pi }}}_{{\rm{study}}}\left(1-{{{\pi }}}_{{\rm{population}}}\right)}{{{{\pi }}}_{{\rm{population}}}\left(1-{{{\pi }}}_{{\rm{study}}}\right)}{\rm{TN}}}.\end{array}$$

### Inclusion and ethics statement

This work was conducted in collaboration with primary care providers serving a diverse patient population. A primary care provider (B.E.) was included as part of the core research team with full access to data, interpretation and authorship of publication. Other primary care providers were provided part-time salary for their efforts in recruitment for the study. This work is part of the NIH-funded Duke Autism Center of Excellence research program (G.D., director), which includes a Dissemination and Outreach Core whose mission is to establish two-way communication with stakeholders related to the center’s research program and includes a Community Engagement Advisory Board comprising autistic self-advocates, parents of autistic children and other key representatives from the broader stakeholder community.

### Reporting summary

Further information on research design is available in the Nature Portfolio Reporting Summary linked to this article.

## Online content

Any methods, additional references, Nature Portfolio reporting summaries, source data, extended data, supplementary information, acknowledgements, peer review information; details of author contributions and competing interests; and statements of data and code availability are available at 10.1038/s41591-023-02574-3.

### Supplementary information

Supplementary Information Supplementary Table 1 and Supplementary Fig. 1.

Reporting Summary

Supplementary Video 1 Short presentation of the app.

Supplementary Video 2 Example of a child playing the game.

## Data Availability

Per National Institutes of Health policy, individual-level descriptive data from this study are deposited in the National Institute of Mental Health National Data Archive (NDA; https://nda.nih.gov) using an NDA Global Unique Identifier (GUID) and made accessible to members of the research community according to provisions defined in the NDA Data Sharing Policy and Duke University Institutional Review Board.

## Code availability

Custom code used in this study is available at: https://github.com/samperochon/Perochon_et_al_Nature_Medicine_2023.
