# Supplementary material for: Early detection of autism using digital behavioral phenotyping
Source: Nat Med. 2023 Oct 2;29(10):2489–97. doi: 10.1038/s41591-023-02574-3 (PMC10579093; doi:10.1038/s41591-023-02574-3)
Supplement: Supplementary file 1 — Supplementary Table 1 and Supplementary Fig. 1. [file 41591_2023_2574_MOESM1_ESM.pdf]

nature medicine

Article https://doi.org/10.1038/s41591-023-02574-3

Early detection of autism using digital behavioral phenotyping

In the format provided by the authors and unedited

Table of contents

Supplementary Information Table S1: Table showing the sensitivity, specificity, and (corrected) Positive Predictive Values (PPV) and Negative Predictive Values (NPV) for different operating points of the Receiver Operating Curve.

Supplementary Information Figure S1. Importance plots of the app variables to the model's prediction.

Supplementary Information Table S1: Table showing the sensitivity, specificity, and (corrected) Positive Predictive Values (PPV) and Negative Predictive Values (NPV) for different operating points of the Receiver Operating Curve. All app variables were used in these analyses, and the model is trained to differentiate autistic (N=49) from neurotypical (N=328) participants. The red and green rows correspond to the operating point that optimizes the Youden and F_2 measures, respectively.

Index threshold	Threshold	Sensitivity (%)	Specificity (%)	PPV (%)	NPV (%)	PPV Adjusted (%)	NPV Adjusted (%)
1	1.0	34.7	98.5	77.3	32.6	91.0	98.6
2	0.999	34.7	97.9	70.8	25.7	90.9	98.6
3	0.998	36.7	97.3	66.7	22.1	91.1	98.6
4	0.997	40.8	97.0	66.7	22.1	91.6	98.7
5	0.996	46.9	97.0	69.7	24.6	92.4	98.9
6	0.989	51.0	97.0	71.4	26.2	93.0	98.9
7	0.986	53.1	96.3	68.4	23.6	93.2	99.0
8	0.961	61.2	96.3	71.4	26.2	94.3	99.2
9	0.957	61.2	96.0	69.8	24.7	94.3	99.1
10	0.954	65.3	96.0	71.1	25.9	94.9	99.2
11	0.951	65.3	95.4	68.1	23.3	94.8	99.2
12	0.935	65.3	94.2	62.7	19.3	94.8	99.2
13	0.932	65.3	93.6	60.4	17.8	94.8	99.2
14	0.899	65.3	92.4	56.1	15.4	94.7	99.2
15	0.898	67.3	92.1	55.9	15.3	95.0	99.3
16	0.895	69.4	92.1	56.7	15.7	95.3	99.3
17	0.879	69.4	91.2	54.0	14.3	95.2	99.3
18	0.858	71.4	91.2	54.7	14.6	95.5	99.3
19	0.848	71.4	90.5	53.0	13.8	95.5	99.3
20	0.834	73.5	90.5	53.7	14.2	95.8	99.4
21	0.777	73.5	89.9	52.2	13.4	95.8	99.4
22	0.775	75.5	89.9	52.9	13.8	96.1	99.4
23	0.765	75.5	89.3	51.4	13.1	96.1	99.4
24	0.755	77.6	89.3	52.1	13.4	96.4	99.5
25	0.657	77.6	86.3	45.8	10.7	96.3	99.5

26	0.654	79.6	86.3	46.4	11.0	96.6	99.5
27	0.653	79.6	86.0	45.9	10.8	96.6	99.5
28	0.65	81.6	86.0	46.5	11.0	96.9	99.5
29	0.607	81.6	83.8	43.0	9.7	96.8	99.5
30	0.579	83.7	83.8	43.6	9.9	97.2	99.6
31	0.471	83.7	82.0	41.0	9.0	97.1	99.6
32	0.466	85.7	82.0	41.6	9.2	97.5	99.6
33 (F_2 optimal)	0.382	85.7	80.8	40.0	8.7	97.4	99.6
34 (Youden optimal)	0.378	87.8	80.8	40.6	8.8	97.8	99.7
35	0.366	87.8	80.2	39.8	8.6	97.8	99.7
36	0.342	87.8	78.7	38.1	8.0	97.7	99.7
37	0.336	89.8	78.7	38.6	8.2	98.1	99.7
38	0.182	89.8	70.7	31.4	6.1	97.9	99.7
39	0.18	89.8	70.1	31.0	6.0	97.9	99.7
40	0.179	91.8	69.8	31.2	6.1	98.3	99.8
41	0.124	91.8	67.7	29.8	5.7	98.2	99.7
42	0.119	91.8	67.1	29.4	5.6	98.2	99.7
43	0.097	91.8	66.5	29.0	5.5	98.2	99.7
44	0.094	91.8	65.9	28.7	5.4	98.2	99.7
45	0.093	91.8	65.5	28.5	5.4	98.2	99.7
46	0.092	93.9	65.5	28.9	5.5	98.6	99.8
47	0.089	93.9	65.2	28.7	5.4	98.6	99.8
48	0.088	93.9	64.3	28.2	5.3	98.6	99.8
49	0.055	93.9	61.3	26.6	4.9	98.5	99.8
50	0.046	93.9	60.7	26.3	4.8	98.5	99.8
51	0.036	93.9	59.5	25.7	4.7	98.5	99.8
52	0.033	93.9	58.8	25.4	4.6	98.5	99.8
53	0.032	93.9	58.5	25.3	4.6	98.5	99.8
54	0.028	93.9	56.7	24.5	4.4	98.4	99.8
55	0.023	93.9	56.4	24.3	4.4	98.4	99.8
56	0.021	93.9	55.8	24.1	4.3	98.4	99.8

57	0.02	93.9	55.5	24.0	4.3	98.4	99.8
58	0.019	93.9	54.9	23.7	4.2	98.4	99.8
59	0.014	93.9	54.0	23.4	4.2	98.3	99.8
60	0.013	93.9	53.4	23.1	4.1	98.3	99.8
61	0.011	93.9	53.0	23.0	4.1	98.3	99.8
62	0.01	95.9	52.4	23.2	4.1	98.9	99.8
63	0.009	95.9	51.5	22.8	4.0	98.8	99.8
64	0.007	95.9	50.9	22.6	4.0	98.8	99.8
65	0.005	96.0	48.0	22.0	99.0	4.0	99.83
66	0.004	96.0	46.0	21.0	99.0	4.0	99.82
67	0.003	96.0	44.0	20.0	99.0	4.0	99.81
68	0.002	96.0	41.0	19.0	99.0	3.0	99.8
69	0.001	96.0	38.0	19.0	98.0	3.0	99.79
70	0.0	100.0	0.0	13.0	100.0	2.0	99.77

Supplementary Information Figure S1. Importance plots of the app variables to the model's prediction. The x-axis represents the variables range, with ticks on the left representing the missing variables. The y-axis represents the normalized SHAP values of that variable. Each point corresponds to the SHAP value of the corresponding variable for each of the participants from the neurotypical and autistic groups. This allows us to understand which ranges of the variables' values correspond to an increase or decrease of the model's prediction towards the autistic or neurotypical groups. SHAP values of participants #1 and #2, presented in the manuscript, are shown with large purple and red dots, and participants #3, #4, and #5, presented in the Extended Data Figure 3, are reported in grey, green, and sky-blue dots, respectively. The app variables are ordered by their global importance to the model.

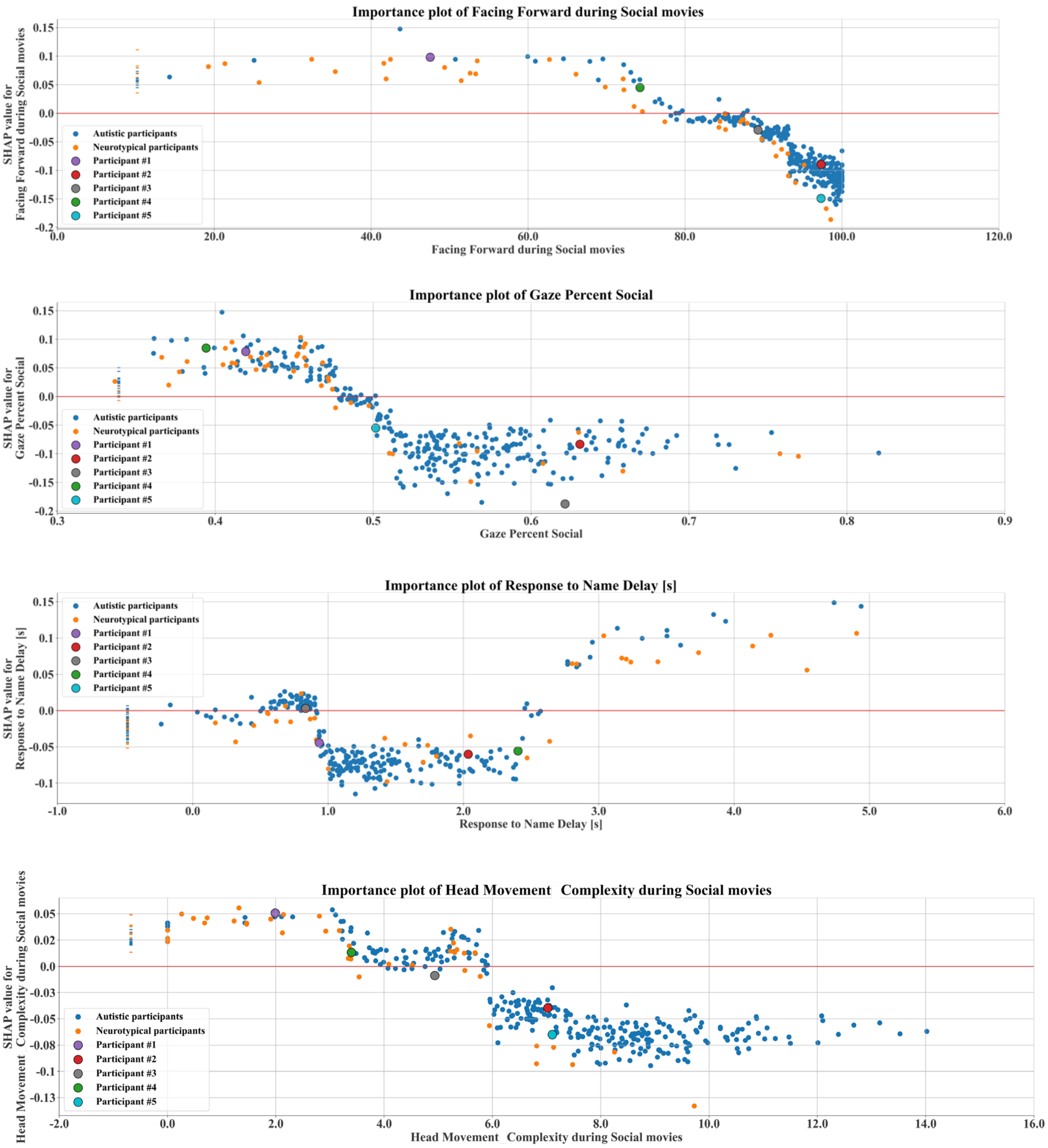

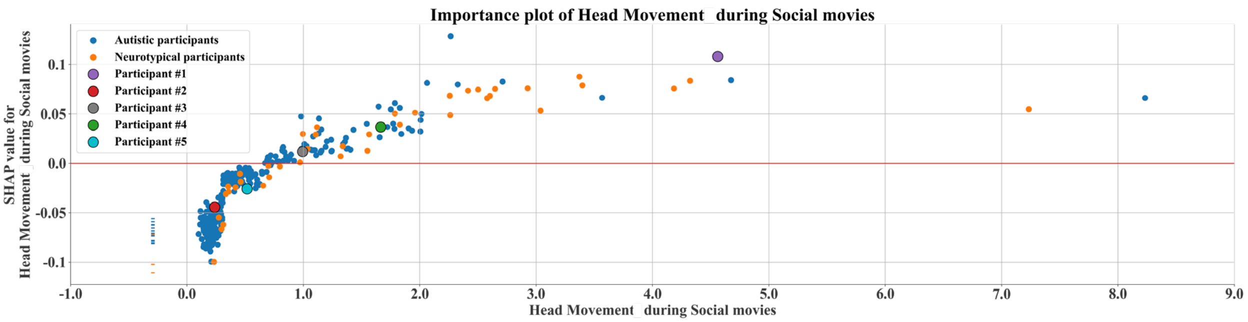
Importance plot of Head Movement during Social movies
SHAP value for
Head Movement during Social movies
Head Movement during Social movies
Autistic participants
Neurotypical participants
Participant #1
Participant #2
Participant #3
Participant #4
Participant #5

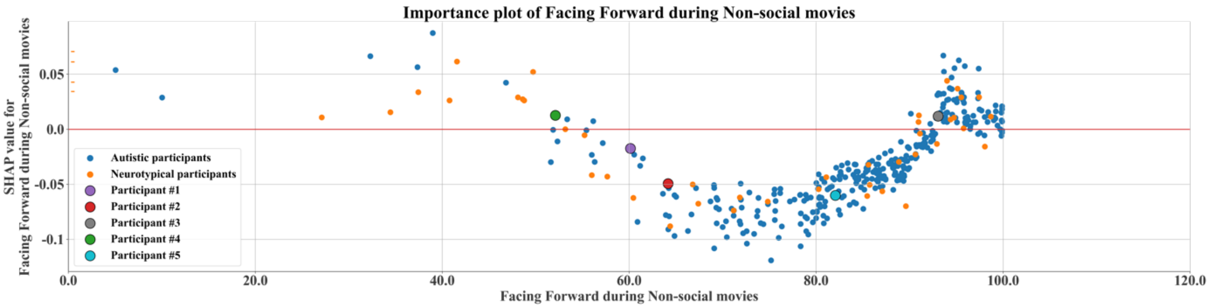
Importance plot of Facing Forward during Non-social movies
SHAP value for
Facing Forward during Non-social movies
Facing Forward during Non-social movies
Autistic participants
Neurotypical participants
Participant #1
Participant #2
Participant #3
Participant #4
Participant #5

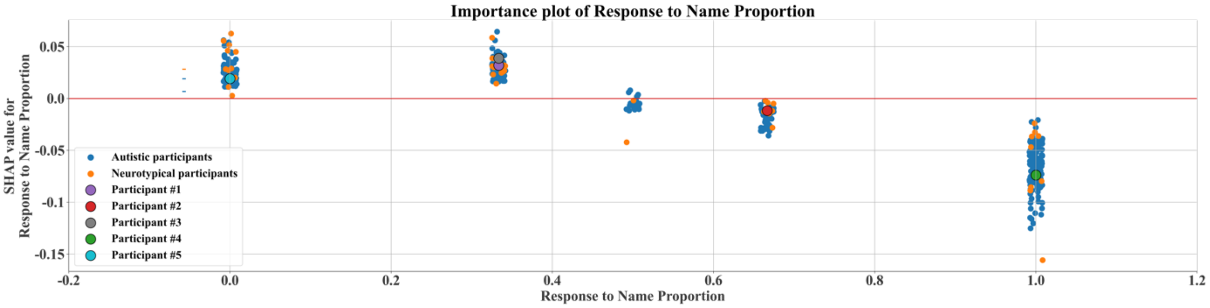
Importance plot of Response to Name Proportion
SHAP value for
Response to Name Proportion
Response to Name Proportion
Autistic participants
Neurotypical participants
Participant #1
Participant #2
Participant #3
Participant #4
Participant #5

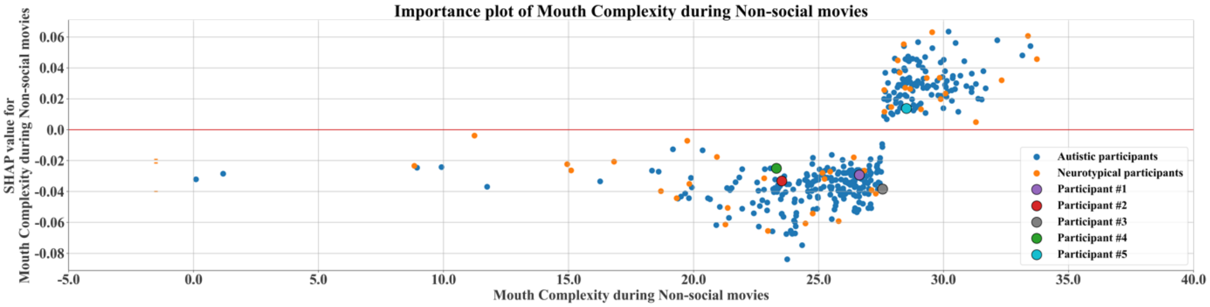
Importance plot of Mouth Complexity during Non-social movies
SHAP value for
Mouth Complexity during Non-social movies
Mouth Complexity during Non-social movies
Autistic participants
Neurotypical participants
Participant #1
Participant #2
Participant #3
Participant #4
Participant #5

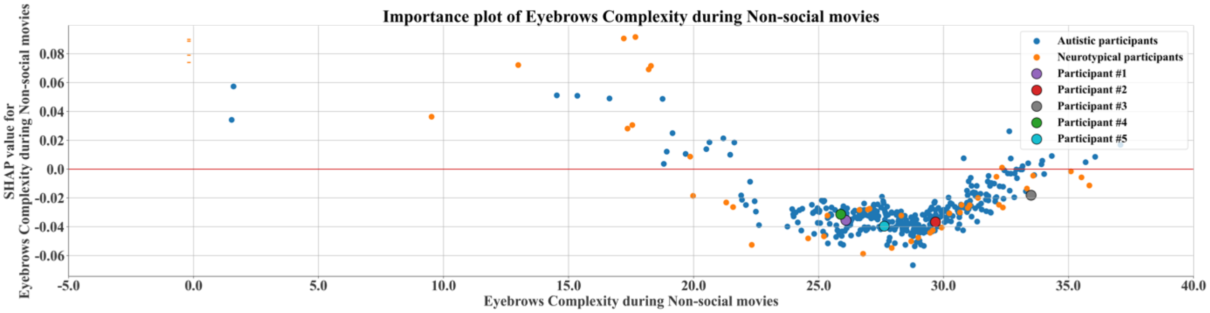
Importance plot of Eyebrows Complexity during Non-social movies
SHAP value for
Eyebrows Complexity during Non-social movies
Eyebrows Complexity during Non-social movies
Autistic participants
Neurotypical participants
Participant #1
Participant #2
Participant #3
Participant #4
Participant #5

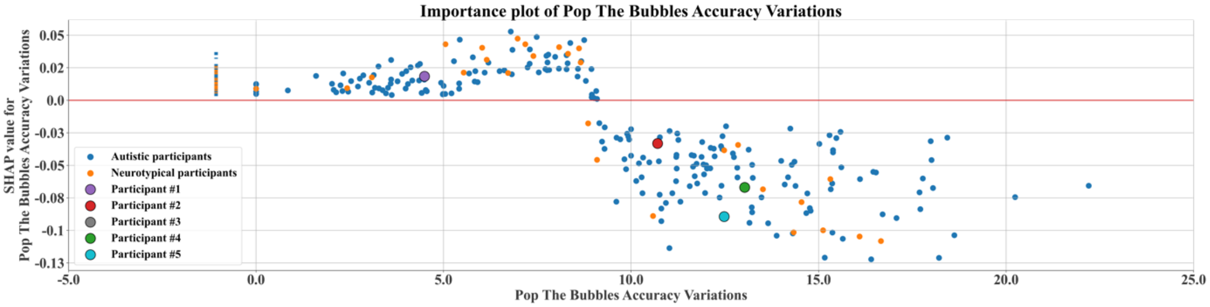
Importance plot of Pop The Bubbles Accuracy Variations
SHAP value for Pop The Bubbles Accuracy Variations
Pop The Bubbles Accuracy Variations
0.05
0.02
0.0
-0.03
-0.05
-0.08
-0.1
-0.13
-5.0
0.0
5.0
10.0
15.0
20.0
25.0
Autistic participants
Neurotypical participants
Participant #1
Participant #2
Participant #3
Participant #4
Participant #5

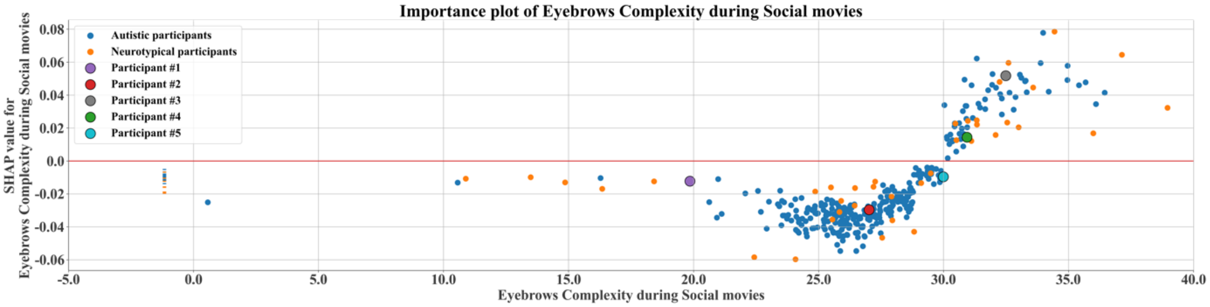
Importance plot of Eyebrows Complexity during Social movies
SHAP value for Eyebrows Complexity during Social movies
Eyebrows Complexity during Social movies
0.08
0.06
0.04
0.02
0.0
-0.02
-0.04
-0.06
-5.0
0.0
5.0
10.0
15.0
20.0
25.0
30.0
35.0
40.0
Autistic participants
Neurotypical participants
Participant #1
Participant #2
Participant #3
Participant #4
Participant #5

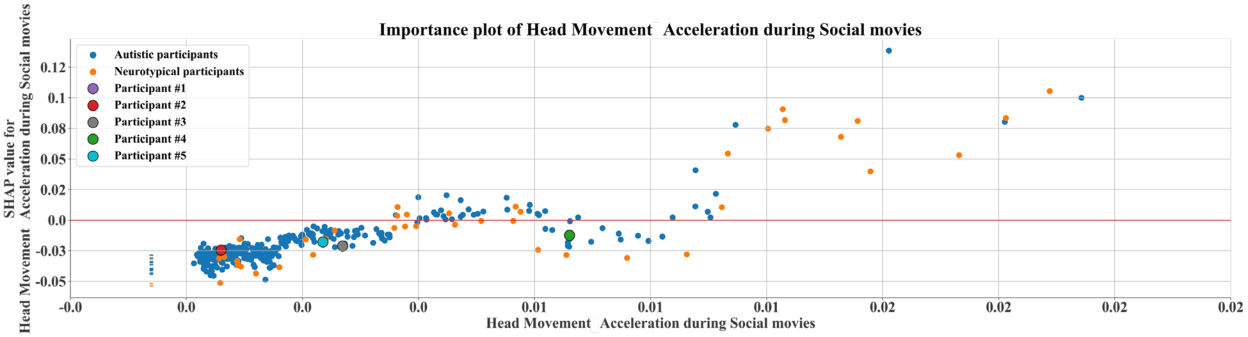
Importance plot of Head Movement Acceleration during Social movies
SHAP value for Head Movement Acceleration during Social movies
Head Movement Acceleration during Social movies
0.12
0.1
0.08
0.05
0.02
0.0
-0.03
-0.05
-0.0
0.0
0.0
0.0
0.01
0.01
0.01
0.02
0.02
0.02
0.02
Autistic participants
Neurotypical participants
Participant #1
Participant #2
Participant #3
Participant #4
Participant #5

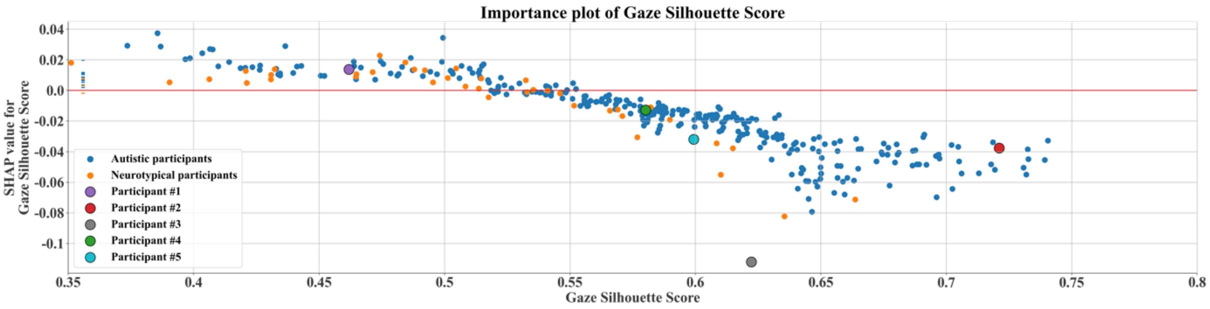
Importance plot of Gaze Silhouette Score
SHAP value for Gaze Silhouette Score
Gaze Silhouette Score
0.04
0.02
0.0
-0.02
-0.04
-0.06
-0.08
-0.1
0.35
0.4
0.45
0.5
0.55
0.6
0.65
0.7
0.75
0.8
Autistic participants
Neurotypical participants
Participant #1
Participant #2
Participant #3
Participant #4
Participant #5

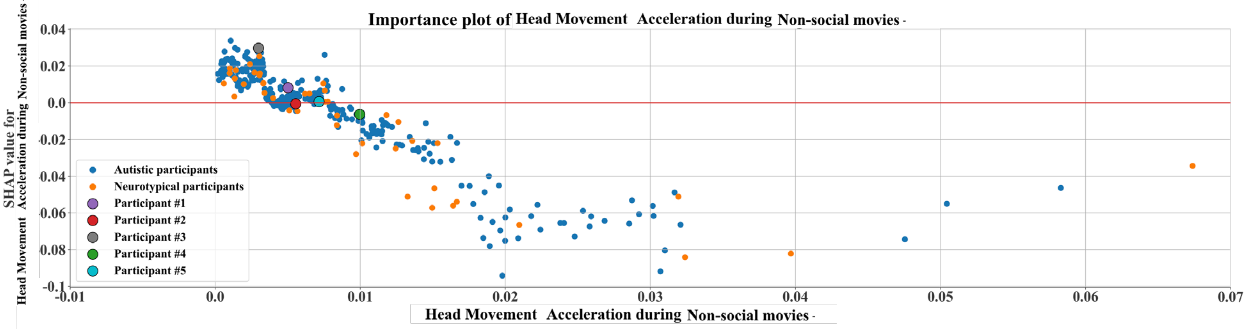
Importance plot of Head Movement Acceleration during Non-social movies
SHAP value for Head Movement Acceleration during Non-social movies
Head Movement Acceleration during Non-social movies
0.04
0.02
0.0
-0.02
-0.04
-0.06
0.08
-0.1
-0.01
0.0
0.01
0.02
0.03
0.04
0.05
0.06
0.07
Autistic participants
Neurotypical participants
Participant #1
Participant #2
Participant #3
Participant #4
Participant #5

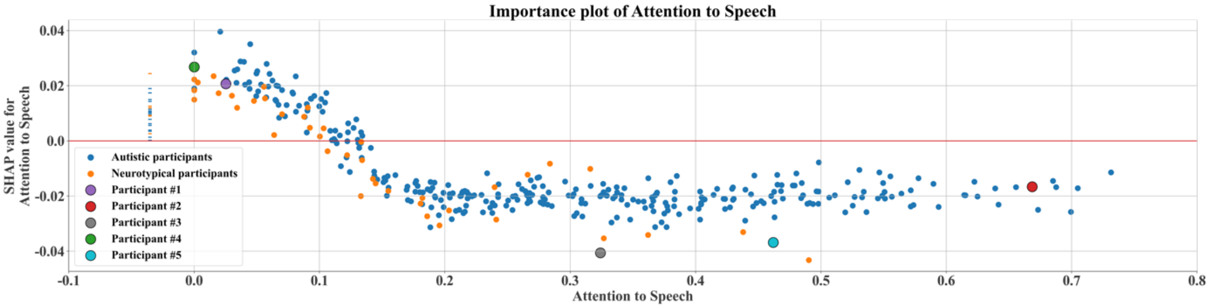
Importance plot of Attention to Speech
SHAP value for Attention to Speech
Attention to Speech
Autistic participants
Neurotypical participants
Participant #1
Participant #2
Participant #3
Participant #4
Participant #5

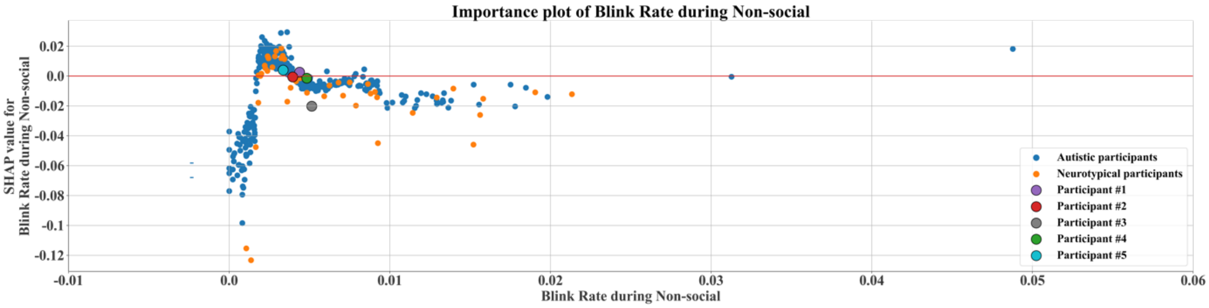
Importance plot of Blink Rate during Non-social
SHAP value for Blink Rate during Non-social
Blink Rate during Non-social
Autistic participants
Neurotypical participants
Participant #1
Participant #2
Participant #3
Participant #4
Participant #5

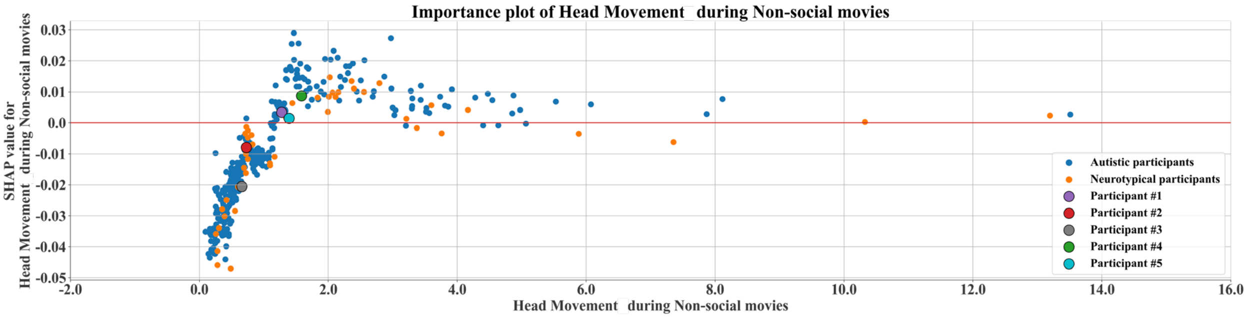
Importance plot of Head Movement during Non-social movies
SHAP value for Head Movement during Non-social movies
Head Movement during Non-social movies
Autistic participants
Neurotypical participants
Participant #1
Participant #2
Participant #3
Participant #4
Participant #5

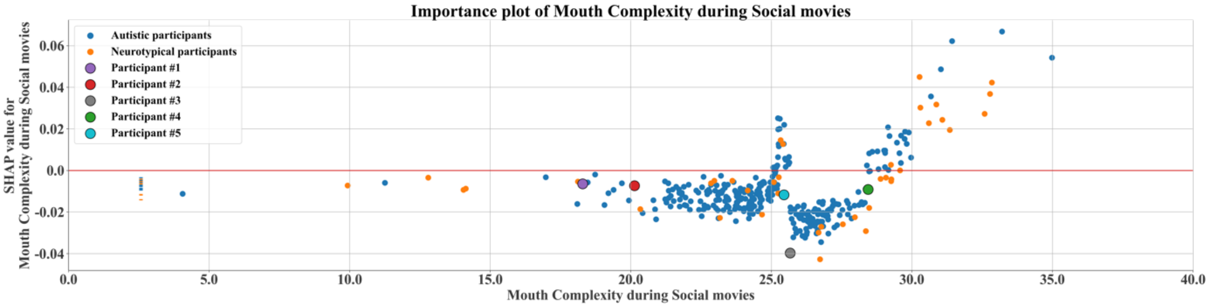
Importance plot of Mouth Complexity during Social movies
SHAP value for Mouth Complexity during Social movies
Mouth Complexity during Social movies
Autistic participants
Neurotypical participants
Participant #1
Participant #2
Participant #3
Participant #4
Participant #5

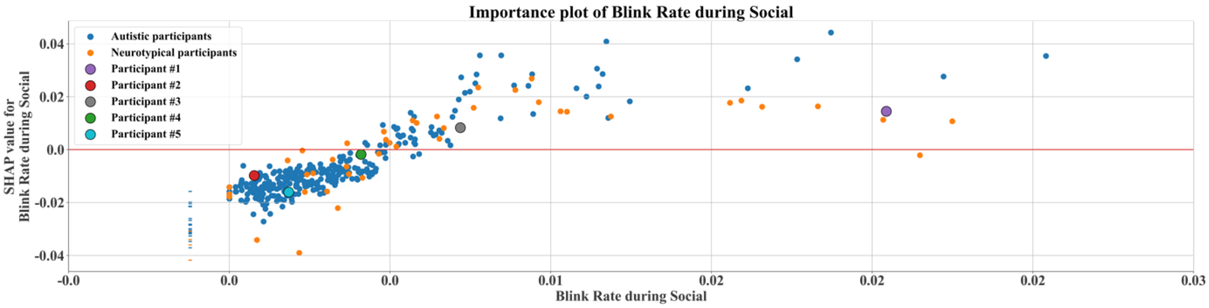
Importance plot of Blink Rate during Social
SHAP value for Blink Rate during Social
Blink Rate during Social
Autistic participants
Neurotypical participants
Participant #1
Participant #2
Participant #3
Participant #4
Participant #5

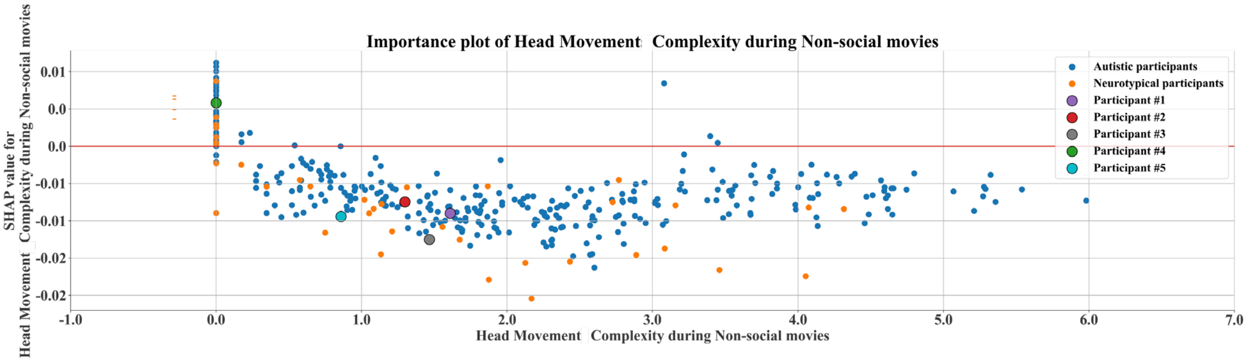
Importance plot of Head Movement Complexity during Non-social movies
SHAP value for Head Movement Complexity during Non-social movies
Head Movement Complexity during Non-social movies
Autistic participants
Neurotypical participants
Participant #1
Participant #2
Participant #3
Participant #4
Participant #5

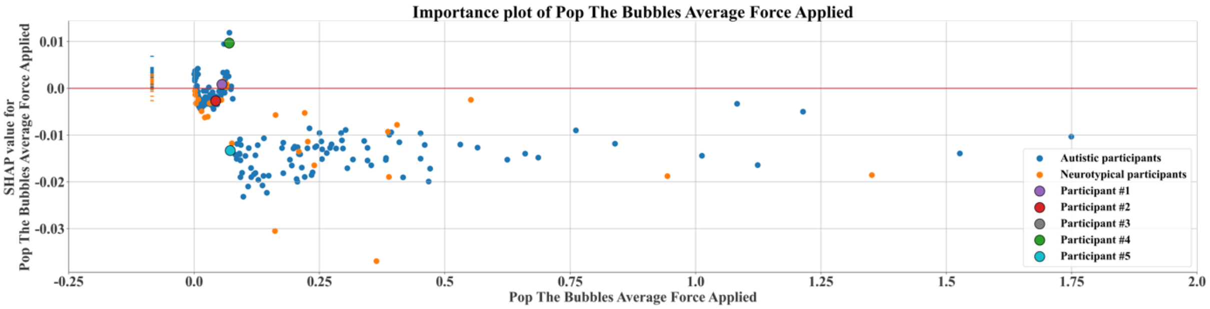
Importance plot of Pop The Bubbles Average Force Applied
SHAP value for Pop The Bubbles Average Force Applied
Pop The Bubbles Average Force Applied
Autistic participants
Neurotypical participants
Participant #1
Participant #2
Participant #3
Participant #4
Participant #5

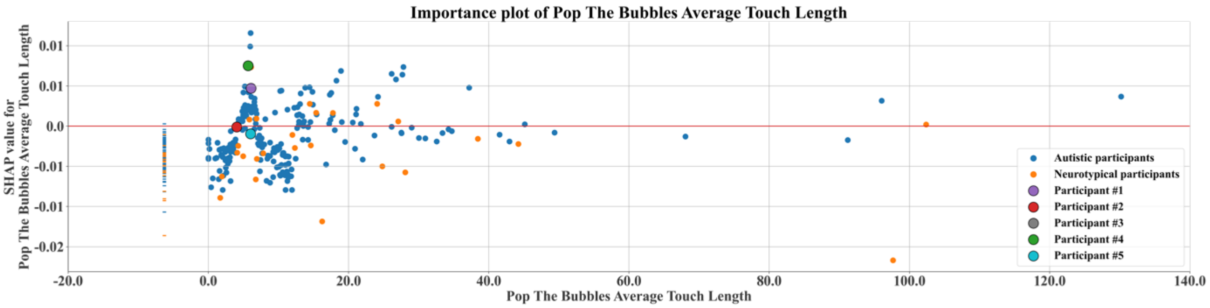
Importance plot of Pop The Bubbles Average Touch Length
SHAP value for Pop The Bubbles Average Touch Length
Pop The Bubbles Average Touch Length
Autistic participants
Neurotypical participants
Participant #1
Participant #2
Participant #3
Participant #4
Participant #5

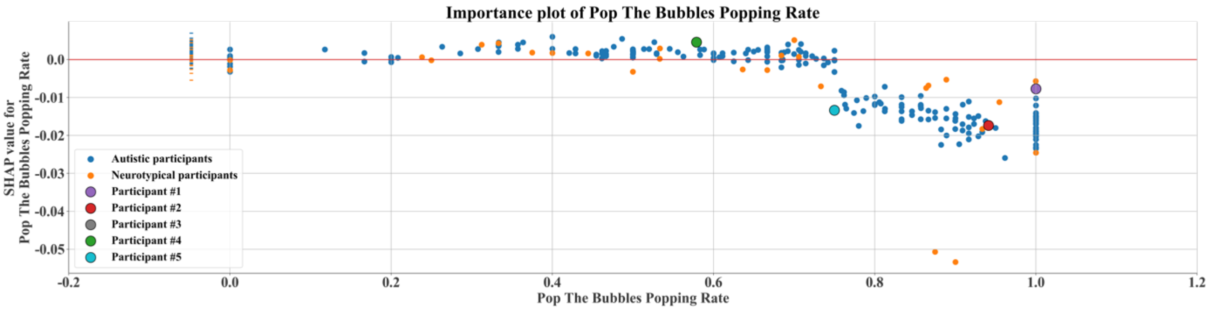
Importance plot of Pop The Bubbles Popping Rate
SHAP value for Pop The Bubbles Popping Rate
Pop The Bubbles Popping Rate
Autistic participants
Neurotypical participants
Participant #1
Participant #2
Participant #3
Participant #4
Participant #5
