# Supplementary material for: Early detection of autism using digital behavioral phenotyping
Source: Nat Med. 2023 Oct 2;29(10):2489–97. doi: 10.1038/s41591-023-02574-3 (PMC10579093; doi:10.1038/s41591-023-02574-3)
Supplement: Supplementary file 2 — Reporting Summary [file 41591_2023_2574_MOESM2_ESM.pdf]

nature portfolio

Corresponding author(s): Geraldine Dawson

Last updated by author(s): 08-21-2023

Reporting Summary

Nature Portfolio wishes to improve the reproducibility of the work that we publish. This form provides structure for consistency and transparency in reporting. For further information on Nature Portfolio policies, see our Editorial Policies and the Editorial Policy Checklist.

Please do not complete any field with "not applicable" or n/a. Refer to the help text for what text to use if an item is not relevant to your study.
For final submission: please carefully check your responses for accuracy; you will not be able to make changes later.

Statistics

For all statistical analyses, confirm that the following items are present in the figure legend, table legend, main text, or Methods section.

n/a	Confirmed	
☐	☑	The exact sample size (*n*) for each experimental group/condition, given as a discrete number and unit of measurement
☐	☑	A statement on whether measurements were taken from distinct samples or whether the same sample was measured repeatedly
☐	☑	The statistical test(s) used AND whether they are one- or two-sided<br>*Only common tests should be described solely by name; describe more complex techniques in the Methods section.*
☐	☑	A description of all covariates tested
☐	☑	A description of any assumptions or corrections, such as tests of normality and adjustment for multiple comparisons
☐	☑	A full description of the statistical parameters including central tendency (e.g. means) or other basic estimates (e.g. regression coefficient) AND variation (e.g. standard deviation) or associated estimates of uncertainty (e.g. confidence intervals)
☐	☑	For null hypothesis testing, the test statistic (e.g. *F*, *t*, *r*) with confidence intervals, effect sizes, degrees of freedom and *P* value noted<br>*Give P values as exact values whenever suitable.*
☐	☑	For Bayesian analysis, information on the choice of priors and Markov chain Monte Carlo settings
☐	☑	For hierarchical and complex designs, identification of the appropriate level for tests and full reporting of outcomes
☐	☑	Estimates of effect sizes (e.g. Cohen's *d*, Pearson's *r*), indicating how they were calculated

Our web collection on statistics for biologists contains articles on many of the points above.

Software and code

Policy information about availability of computer code

Data collection	Data were captured using REDCap software versions 8.1, 8.5, 8.10, 9.1, 9.5, 10.0, 10.6, and 12.0.
Data analysis	Statistics were calculated in Python V.3.8.10, using SciPy low-level functions V.1.7.3, XGBoost and SHAP official implementations V.1.5.2 and V.0.40.0, respectively.<br>Custom code that supports the findings of this study is available at the following location: https://github.com/samperochon/Perochon_et_al_Nature_Medicine_2023.

For manuscripts utilizing custom algorithms or software that are central to the research but not yet described in published literature, software must be made available to editors and reviewers. We strongly encourage code deposition in a community repository (e.g. GitHub). See the Nature Portfolio guidelines for submitting code & software for further information.

Data

Policy information about availability of data

All manuscripts must include a data availability statement. This statement should provide the following information, where applicable:

- Accession codes, unique identifiers, or web links for publicly available datasets
- A description of any restrictions on data availability
- For clinical datasets or third party data, please ensure that the statement adheres to our policy

As required by the National Institutes of Health, individual-level descriptive data from this study are deposited in the National Institute of Mental Health National Data Archive (NDA) using an NDA Global Unique Identifier (GUID) and made accessible to members of the research community according to provisions defined in the NDA Data Sharing Policy and Duke University Institutional Review Board.

Research involving human participants, their data, or biological material

Policy information about studies with human participants or human data. See also policy information about sex, gender (identity/presentation), and sexual orientation and race, ethnicity and racism.

Reporting on sex and gender: 269 boys; 206 girls

Reporting on race, ethnicity, or other socially relevant groupings: 425 Not Hispanic/Latino; 50 Hispanic/Latino; 4 American Indian/Alaska Native; 7 Asian; 54 Black or African American; 47 More than one race reported; 15 Not reported/Other

Population characteristics: Participants were patients at one of four Duke University Health System pediatrics primary care clinics who were 17-36 months of age and did not have significant sensory or motor impairments, were not ill, and whose parents spoke English or Spanish. Of the 475 participants, 49 were diagnosed with autism spectrum disorder, 98 with developmental or language delay without autism, and 328 were considered to have neurotypical development.

Recruitment: Parents or legal guardians of potential participants were approached by study staff during their child's well-child visit to a Duke University Health System (DUHS) pediatric primary care clinic and invited to participate in the present study. The clinic population roughly matches that of Durham, NC; approximately 86% of children living in Durham County, North Carolina, receive their primary care within the DUHS. Potential biases include exclusion of children with sensory and/or motor impairments and those whose parents did not speak English or Spanish. Racial and ethnic diversity of enrolled participants was greater for participants diagnosed with autism or developmental/language delay than for those with neurotypical development, with the clinical groups more closely matching the ethnic and racial distribution of the DUHS and Durham County, NC.

Ethics oversight: Duke University Institutional Review Board

Note that full information on the approval of the study protocol must also be provided in the manuscript.

Field-specific reporting

Please select the one below that is the best fit for your research. If you are not sure, read the appropriate sections before making your selection.

☐ Life sciences ☑ Behavioural & social sciences ☐ Ecological, evolutionary & environmental sciences

For a reference copy of the document with all sections, see nature.com/documents/nr-reporting-summary-flat.pdf

Life sciences study design

All studies must disclose on these points even when the disclosure is negative.

Sample size:

Data exclusions:

Replication:

Randomization:

Blinding:

Behavioural & social sciences study design

All studies must disclose on these points even when the disclosure is negative.

Study description: Prospective, non-experimental study design based on quantitative data.

Research sample: The research sample was chosen based on the intended use of the SenseToKnow app as an autism screening tool administered as part of a child's routine 18-24 month well child visit in pediatric primary care. Participants were representative of patients at one of four Duke University Health System (DUHS) pediatrics primary care clinics who were 17-36 months of age and did not have significant sensory or motor impairments, were not ill, and whose parents spoke English or Spanish. Racial and ethnic diversity of enrolled participants was greater for participants diagnosed with autism or developmental/language delay than for those with neurotypical development, with the clinical groups more closely matching the ethnic and racial distribution of the DUHS and Durham County, NC.

Sampling strategy: Consecutive recruitment and enrollment of Duke University Health System patients in pediatric primary care clinics and sample size providing adequate statistical power to test of the hypothesis that the sensitivity and specificity of the SenseToKnow app for autism detection relative to expert clinical diagnosis are > 70% (alpha=0.05).

Data collection: Data were collected during a well-child visit to primary care. Parents held their child on their lap while brief, engaging movies were presented on an iPad set on a tripod approximately 60 cm away from the child. Parents were asked to refrain from talking during the movies. The frontal camera embedded in the device recorded the child's behavior at resolutions of 1280 × 720, 30 frames per second. While children were watching the movies, their name was called three times by an examiner standing behind them at pre-defined timestamps. The children then participated in a game using their finger to pop a set of colored bubbles that moved continuously across the screen. App completion took <10 minutes. Study staff responsible for app administration were blind to the child's diagnosis and clinicians resp onsible for making the child's clinical diagnosis were blind to the SenseToKnow app's diagnostic classification.

Timing: The study was conducted from December 2018 to March 2020.

Data exclusions: No data excluded.

Non-participation: 754 patients invited to participate; 214 declined; 513 eligible and consented; 475 (93% of patients enrolled) completed study measures.

Randomization:

Diagnostic classification was made naive to results of the autism screening app results. Children were administered the Modified Checklist for Autism in Toddlers (M-CHAT-R/F), a parent survey querying different autism signs. Children with a final M-CHAT-R/F score of >2 or whose parents and/or provider expressed any developmental concern were provided a gold standard autism diagnostic evaluation based on the Autism Diagnostic Observation Schedule–Second Edition (ADOS-2),2 DSM-5 criteria checklist, and Mullen Scales of Early Learning,3 conducted by a licensed, research-reliable psychologist who was blind with respect to app results. Mean duration between app screening and evaluation = 3.5 months, which is a similar or shorter duration compared to real-world settings. Diagnosis of autism spectrum disorder required meeting full DSM-5 diagnostic criteria. Diagnosis of developmental or language delay without autism (DD-LD) was defined as failing the M-CHAT-R/F and/or having provider or parent concerns and having been administered the ADOS-2 and Mullen Scales and determined by the psychologist not to meet diagnostic criteria for autism and exhibiting developmental and/or language delay based on the Mullen Scales (scoring > 9 points below the mean on at least one Mullen Scales subscale; SD=10).

In addition, each participant's Duke University Health System electronic health record (EHR) was monitored through age 4 years to confirm whether the child subsequently received a diagnosis of either autism spectrum disorder or DD-LD. Following validated methods used by Guthrie et al., children were classified as autistic or DD-LD based on their EHR record if an ICD-9/10 diagnostic code for autism spectrum disorder or DD-LD (without autism) appeared more than once or was provided by an autism specialty clinic. 4 If a child did not have an elevated M-CHAT-R/F score, no developmental concerns were raised by the provider or parents, and there were no autism or DD-LD diagnostic codes in the EHR through age four, they were considered neurotypical. There were 2 children classified as neurotypical who scored positive on the M-CHAT-R/F who were considered neurotypical based on expert diagnostic evaluation and had no autism or DD-LD EHR diagnostic codes.
Based on these procedures, 49 children were diagnosed with autism spectrum disorder (6 based on EHR only), 98 children were diagnosed DD-LD without autism (78 based on EHR only), and 328 children were considered neurotypical.

Ecological, evolutionary & environmental sciences study design

All studies must disclose on these points even when the disclosure is negative.

Study description

Research sample

Sampling strategy

Data collection

Timing and spatial scale

Data exclusions

Reproducibility

Randomization

Blinding

Did the study involve field work? ☐ Yes ☐ No

Field work, collection and transport

Field conditions

Location

Access & import/export

Disturbance

Reporting for specific materials, systems and methods

We require information from authors about some types of materials, experimental systems and methods used in many studies. Here, indicate whether each material, system or method listed is relevant to your study. If you are not sure if a list item applies to your research, read the appropriate section before selecting a response.

Materials & experimental systems

n/a	Involved in the study
☑	☐ Antibodies
☑	☐ Eukaryotic cell lines
☑	☐ Palaeontology and archaeology
☑	☐ Animals and other organisms
☑	☐ Clinical data
☑	☐ Dual use research of concern
☑	☐ Plants

Methods

n/a	Involved in the study
☑	☐ ChIP-seq
☑	☐ Flow cytometry
☑	☐ MRI-based neuroimaging

Antibodies

Antibodies used

Validation

Eukaryotic cell lines

Policy information about cell lines and Sex and Gender in Research

Cell line source(s)	
Authentication	
Mycoplasma contamination	
Commonly misidentified lines (See ICLAC register)	

Palaeontology and Archaeology

Specimen provenance	
Specimen deposition	
Dating methods	

☐ Tick this box to confirm that the raw and calibrated dates are available in the paper or in Supplementary Information.

Ethics oversight	

Note that full information on the approval of the study protocol must also be provided in the manuscript.

Animals and other research organisms

Policy information about studies involving animals; ARRIVE guidelines recommended for reporting animal research, and Sex and Gender in Research

Laboratory animals	
Wild animals	
Reporting on sex	
Field-collected samples	
Ethics oversight	

Note that full information on the approval of the study protocol must also be provided in the manuscript.

Clinical data

Policy information about clinical studies
All manuscripts should comply with the ICMJE guidelines for publication of clinical research and a completed CONSORT checklist must be included with all submissions.

Clinical trial registration	DUHSPro00085434
Study protocol	Duke University Protocol # Pro00085434
Data collection	Data was collected in Duke Primary Care pediatric clinics from December 2018 through March 2020.
Outcomes	Outcome was a diagnostic classification of autism spectrum disorder (DSM-5 criteria), language or developmental delay without autism, or neurotypical development as assessed via expert clinical evaluation and/or diagnostic codes in the patient's electronic health record.

Dual use research of concern

Policy information about dual use research of concern

Hazards

Could the accidental, deliberate or reckless misuse of agents or technologies generated in the work, or the application of information presented in the manuscript, pose a threat to:

No | Yes

- [ ] [ ] Public health
- [ ] [ ] National security
- [ ] [ ] Crops and/or livestock
- [ ] [ ] Ecosystems
- [ ] [ ] Any other significant area

Experiments of concern

Does the work involve any of these experiments of concern:

No | Yes

- [ ] [ ] Demonstrate how to render a vaccine ineffective
- [ ] [ ] Confer resistance to therapeutically useful antibiotics or antiviral agents
- [ ] [ ] Enhance the virulence of a pathogen or render a nonpathogen virulent
- [ ] [ ] Increase transmissibility of a pathogen
- [ ] [ ] Alter the host range of a pathogen
- [ ] [ ] Enable evasion of diagnostic/detection modalities
- [ ] [ ] Enable the weaponization of a biological agent or toxin
- [ ] [ ] Any other potentially harmful combination of experiments and agents

Plants

Seed stocks

Novel plant genotypes

Authentication

ChIP-seq

Data deposition

- [ ] Confirm that both raw and final processed data have been deposited in a public database such as GEO.
- [ ] Confirm that you have deposited or provided access to graph files (e.g. BED files) for the called peaks.

Data access links
May remain private before publication.

Files in database submission

Genome browser session
(e.g. UCSC)

Methodology

Replicates

Sequencing depth

Antibodies

Peak calling parameters

Data quality

Software

Flow Cytometry

Plots

Confirm that:

☐ The axis labels state the marker and fluorochrome used (e.g. CD4-FITC).

☐ The axis scales are clearly visible. Include numbers along axes only for bottom left plot of group (a 'group' is an analysis of identical markers).

☐ All plots are contour plots with outliers or pseudocolor plots.

☐ A numerical value for number of cells or percentage (with statistics) is provided.

Methodology

Sample preparation

Instrument

Software

Cell population abundance

Gating strategy

☐ Tick this box to confirm that a figure exemplifying the gating strategy is provided in the Supplementary Information.

Magnetic resonance imaging

Experimental design

Design type

Design specifications

Behavioral performance measures

Imaging type(s)

Field strength

Sequence & imaging parameters

Area of acquisition

Diffusion MRI ☐ Used ☐ Not used

Preprocessing

Preprocessing software

Normalization

Normalization template

Noise and artifact removal

Volume censoring

Statistical modeling & inference

Model type and settings

Effect(s) tested

Specify type of analysis: ☐ Whole brain ☐ ROI-based ☐ Both

Statistic type for inference

(See Eklund et al. 2016)

Correction

Models & analysis

n/a	Involved in the study
☐	☐ Functional and/or effective connectivity
☐	☐ Graph analysis
☐	☐ Multivariate modeling or predictive analysis

Functional and/or effective connectivity

Graph analysis

Multivariate modeling and predictive analysis

This checklist template is licensed under a Creative Commons Attribution 4.0 International License, which permits use, sharing, adaptation, distribution and reproduction in any medium or format, as long as you give appropriate credit to the original author(s) and the source, provide a link to the Creative Commons license, and indicate if changes were made. The images or other third party material in this article are included in the article's Creative Commons license, unless indicated otherwise in a credit line to the material. If material is not included in the article's Creative Commons license and your intended use is not permitted by statutory regulation or exceeds the permitted use, you will need to obtain permission directly from the copyright holder. To view a copy of this license, visit http://creativecommons.org/licenses/by/4.0/
